# Visual system function requires translational regulation of ATF4 by Hbs1-Pelo

**DOI:** 10.1038/s44319-026-00882-6

**Published:** 2026-07-17

**Authors:** Katherine Tempro, Inês Lago-Baldaia, Narayanan Nampoothiri V P, Christopher Garbark, Abby J Carney, Vilaiwan M Fernandes, Deepika Vasudevan

**Affiliations:** 1 Department of Cell Biology, University of Pittsburgh School of Medicine, Pittsburgh, USA; 2 Department of Cell and Developmental Biology, University College London, London, UK

**Keywords:** Development, Signal Transduction, Translation & Protein Quality

## Abstract

Deletion mutations in the translation termination factor HBS1L result in progressive loss of vision in human patients, amongst other developmental anomalies. The etiology of vision defects seen with HBS1L deletion remains unknown. Here, we use the Drosophila visual system to demonstrate that the HBS1L ortholog, Hbs1, and its interaction partner, Pelo, are required for proper phototransduction. Hbs1 mutants showed ‘vacuolization’ of the lamina layer, indicative of defective synapse transmission between photoreceptors and lamina neurons. Depleting Hbs1 in lamina neurons replicated the phototransduction defects seen in Hbs1 mutants, suggesting that Hbs1-Pelo is required for proper lamina neuron function. Mechanistically, we found that loss of HBS1L in both Drosophila and cultured human cells results in reduced levels of the stress responsive Activating Transcription Factor 4 (ATF4). Strikingly, restoring ATF4 expression in the lamina partially rescues ERG defects in Hbs1 mutants, indicating that ATF4 is likely a relevant mRNA target regulated by Hbs1-Pelo in these cells. Together, we propose a model wherein Hbs1-Pelo-mediated translation regulation of ATF4 in lamina neurons underlies the inherited retinal disease caused by HBS1L deletion.

## Synopsis

The ribosome recycling complex Hbs1-Pelo regulates expression of the stress response transcription factor ATF4 in the visual system. Hbs1-Pelo facilitates termination at uORFs in the ATF4 5’ leader, thereby promoting efficient ATF4 ORF translation.

- The ribosome recycling complex Hbs1-Pelo regulates ATF4 translation in *Drosophila* and human cells.
- Hbs1 and Pelo are required for efficient translation termination at upstream ORFs in the ATF4 5’ leader.
- In *Drosophila*, Hbs1-Pelo is required in lamina neurons, which interface with photoreceptors for proper phototransduction.
- Restoring ATF4 in lamina neurons partially rescues phototransduction defects seen in Hbs1 mutants.

## Abstract (web summary)

The ribosome recycling complex Hbs1-Pelo regulates expression of the stress response transcription factor ATF4 in the visual system. Hbs1-Pelo facilitates termination at uORFs in the ATF4 5’ leader, thereby promoting efficient ATF4 ORF translation.

## Graphical abstract

**Figure:**
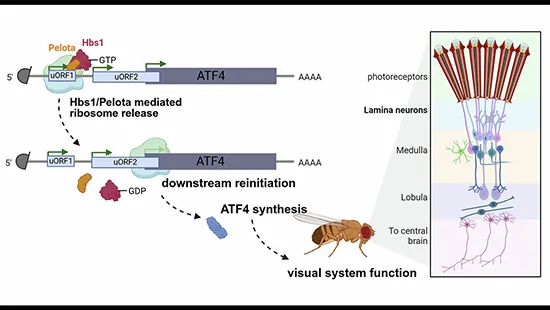

**Figure:**
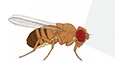

## Introduction

Organisms have evolved an array of stress response pathways to counteract a wide spectrum of extrinsic and intrinsic stressors. In higher organisms, these protective mechanisms have been co-opted to play essential roles in normal tissue development and homeostasis, ensuring that cells adapt and function optimally under varying physiological conditions. A classic example of such a mechanism is the Integrated Stress Response (ISR), an evolutionarily conserved pathway initiated by stress-responsive kinases. ISR signaling famously regulates the translation landscape by reducing global translation by limiting initiator methionine availability (Ryoo and Vasudevan, 2017). These limiting conditions, however, promote the translation of stress-responsive mRNAs with a specific 5’ leader architecture, ATF4 (Activating Transcription Factor 4) being a prime example (Ryoo and Vasudevan, 2017). Loss of ATF4 has been linked to a plethora of developmental abnormalities in the skeletal, hematopoetic, and visual systems, and in the normal functioning adipose and liver tissues. While we have a fair understanding of many ATF4 phenotypes, the role of ATF4 in visual system development is relatively understudied.

The lens in the eye focuses light onto the retina, which is comprised of several types of highly specialized neurons that are organized in distinct stratified layers. Phototransduction is initiated in photoreceptors where light is sensed by opsin proteins which trigger a change in membrane potential. This change in membrane potential modulates neurotransmitter release, sending visual information to downstream neurons. Photoreceptors form synapses with bipolar neurons (Sanes and Zipursky, 2010), amongst others, which then transduce the signal on to ganglion cells, whose axons form the optic nerve. Several studies have shown that loss of ATF4 results in developmental abnormalities in the lens in mice (Nandakumar et al, 2025). ATF4 expression is also robustly detected in developing photoreceptors (Ooe et al, 2017; Kang et al, 2015; Vasudevan et al, 2022; Preston et al, 2025) and is also elevated in the aging retina (Ooe et al, 2017; Vasudevan et al, 2022). In addition to its role in lens development in mice, we previously demonstrated that *ATF4* mutants display age-dependent retinal degeneration in *Drosophila* (Vasudevan et al, 2022). ATF4 has also been implicated in the progression of many retinal degeneration diseases including autosomal dominant retinitis pigmentosa, diabetic retinopathy, and Fuchs’ endothelial corneal dystrophy amongst others (Bhootada et al, 2016; Pitale et al, 2017; Vasudevan et al, 2022; Qureshi et al, 2024). Given these profound developmental and clinical implications, there is significant interest in identifying novel regulators of ATF4 and delineating the specific visual system cell types critically dependent on ISR signaling for their normal development and function.

The ATF4 mRNA is almost ubiquitously detected in mice and *Drosophila* tissues (Leader et al, 2018; Yang and Karsenty, 2004), with ATF4 protein levels being regulated primarily at the level of mRNA translation (Neill and Masson, 2023). Across phyla, the 5’ leader sequence of ATF4 mRNA contains upstream open reading frames (uORFs) in addition to the main open reading frame which encodes ATF4 protein (Hinnebusch et al, 2016). While the number of these uORFs varies across species, the general regulatory architecture is evolutionarily conserved from yeast to *Drosophila* and humans (Hinnebusch, 1984; Lu et al, 2004; Vattem and Wek, 2004; Kang et al, 2015). Human ATF4, for instance, possesses two uORFs, with the second uORF partially overlapping the ATF4 coding sequence itself (Lu et al, 2004; Vattem and Wek, 2004). It is worth noting here that there are discrepancies in the number of annotated uORFs in the human ATF4 5’ leader, with some studies regarding there to be only two uORFs (Vattem and Wek, 2004; Lu et al, 2004), since they exclude a zero-length (start-stop) ORF that is most distal to the ATF4 start codon (Bohlen et al, 2020; Rendleman et al, 2024; Smirnova et al, 2024). Under homeostatic conditions, translation typically initiates and terminates at uORF1, with only a subset of ribosomes reinitiating at uORF2 (Hinnebusch et al, 2016). However, due to the overlapping nature of uORF2 with the ATF4 coding region, ATF4 protein synthesis does not occur when initiator methionyl-tRNA bound to the initiation factor eIF2 is abundant. Upon stress-induced phosphorylation of the alpha subunit of eIF2 by ISR kinases, the resulting reduction in active eIF2 complex abundance leads to ribosomes skipping initiation at uORF2. Consequently, ribosomes instead reinitiate translation at the main ATF4 ORF, resulting in increased ATF4 synthesis during ISR activation. In addition to being regulated by eIF2 availability as modulated by ISR kinases, translation initiation at the ATF4 ORF has been shown to be dependent on specific initiation factors (Roy et al, 2010; Herrmannová et al, 2024) and 5’ leader features (Chan et al, 2013; Rendleman et al, 2024; Smirnova et al, 2024). However, efficient termination at uORF1 is also a key regulatory step in reinitiation at the ATF4 ORF (Ait Ghezala et al, 2012; Bohlen et al, 2020; Vasudevan et al, 2020).

Termination commences with stop codon recognition, followed by release factor recruitment, peptidyl-tRNA hydrolysis, and finally ribosome dissociation. High-resolution ribosome profiling data have revealed that the tRNA-binding factors, DENR-MCTS1 heterodimer and its homolog eIF2D, efficiently remove spent tRNAs from the penultimate codons in uORF1 (Bohlen et al, 2020). Such removal of spent tRNAs is predictably crucial for proper termination at uORF1, and loss of these factors significantly impaired reinitiation at the ATF4 ORF (Bohlen et al, 2020). These results raise the intriguing possibility that there may be additional such termination factors which regulate other critical steps of translation termination at uORF1. Our work herein substantiates this possibility by discovering that the ribosome recycling factor, HBS1L and its binding partner Pelo, are required for efficient ATF4 translation. We use a *Drosophila* model to describe that such regulation is crucial for proper functioning of the visual system. Importantly, our work proffers ATF4 as a mechanistic target underlying the vision defects and other developmental anomalies seen in human patients with HBS1L deficiency.

## Results

### Identifying Hbs1-Pelo as regulators of *Drosophila* ATF4

Recent work demonstrated that loss of the tRNA-binding protein complex, DENR-MCTS1, results in the accumulation of 40S intermediates at certain penultimate codons, including in the uORF1 of ATF4 mRNA (Bohlen et al, 2020). Based on this, we hypothesized that the action of ribosome recycling factors, whose function is to separate the 40S and 60S subunits, precedes removal of spent tRNA DENR-MCTS1/eIF2D. Since we had previously used the *Drosophila* model to discover that loss of DENR-MCTS1/eIF2D resulted in reduced ATF4 translation, we performed a targeted RNAi screen to test whether known termination and ribosome recycling factors were required specifically for ATF4 translation.

In eukaryotes, the release factor eRF1 (eukaryotic release factor 1) binds to the stop codon, and eRF3, a GTPase, facilitates the release of the newly synthesized polypeptide from the ribosome (Hellen, 2018). The eRF1 and eRF3 proteins subsequently dissociate from the ribosome, and the ribosomal subunits (40S and 60S) are separated by the ATP-binding cassette protein ABCE1 (Hellen, 2018). In addition to these core termination factors, a host of other factors with paralogous function have been identified. Pelota (Pelo) has been shown to substitute for eRF1 function (Atkinson et al, 2008; Pisareva et al, 2011; Hellen, 2018). In mammals, two distinct genes encode two different eRF3 forms, namely eRF3a and eRF3b (Chauvin et al, 2005). The GTPases HBS1L (Hsp70 Subfamily B Suppressor 1-like), GTPBP1 and 2 (GTP-binding protein 1, 2) have likewise been shown to perform functions similar to eRF3 (Atkinson et al, 2008; Pisareva et al, 2011; Ishimura et al, 2014; Terrey et al, 2020).

**Figure 1:**
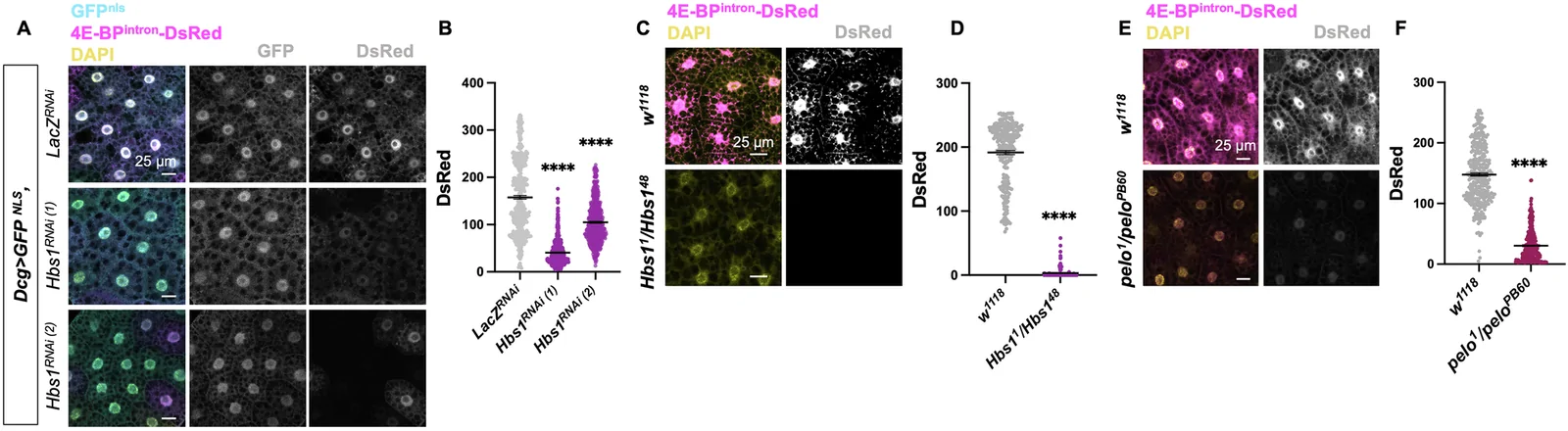
*Hbs1* and *Pelo* are required for ATF4 activity in the *Drosophila* fat body. (**A**) Confocal images showing fat bodies from animals expressing an ATF4 reporter (4E-BP^intron^-DsRed, magenta) and GFP (cyan) driven by *Dcg-GAL4*, which also drives expression of the indicated *RNAi*. DAPI marks nuclei in yellow. Presence of all three markers (DsRed, GFP, and DAPI) renders as white in the multi-channel image; presence of only two markers (GFP, DAPI) renders as green in the multi-channel image. (**B**) Quantification of DsRed intensity from (**A**). Each data point represents one nucleus, and black bar represents mean of data from at least 5–7 animals across two independent crosses. Error bars represent standard error. Statistical comparisons were made using Kruskal–Wallis test with Dunn’s correction. *p*-values are approximate. (**C**,** E**) Confocal images of fat bodies expressing 4E-BP^intron^-DsRed (magenta) from wild type (*w*^*1118*^), and transheterozygous mutants for *Hbs1* (*Hbs1*^*1*^*/Hbs1*^*48*^), and *pelo* (*pelo*^*1*^*/pelo*^*PB60*^). DAPI marks nuclei in yellow. (**D**,** F**) Quantification of DsRed intensity from (**C**) and (**E**), respectively. Each data point represents one nucleus, and black bar represents mean of data from at least 5–7 animals across two independent crosses. Error bars represent standard error of mean. *p*-values were calculated using Mann–Whitney test. *p*-values are approximate. Data information: Scale bars in (**A**), (**C**), (**E**): 25 μm. ****p < 0.0001, ***p < 0.001, **p < 0.01, *p < 0.05, n.s. = not significant. Source data are available online for this figure.

**Figure EV1:**
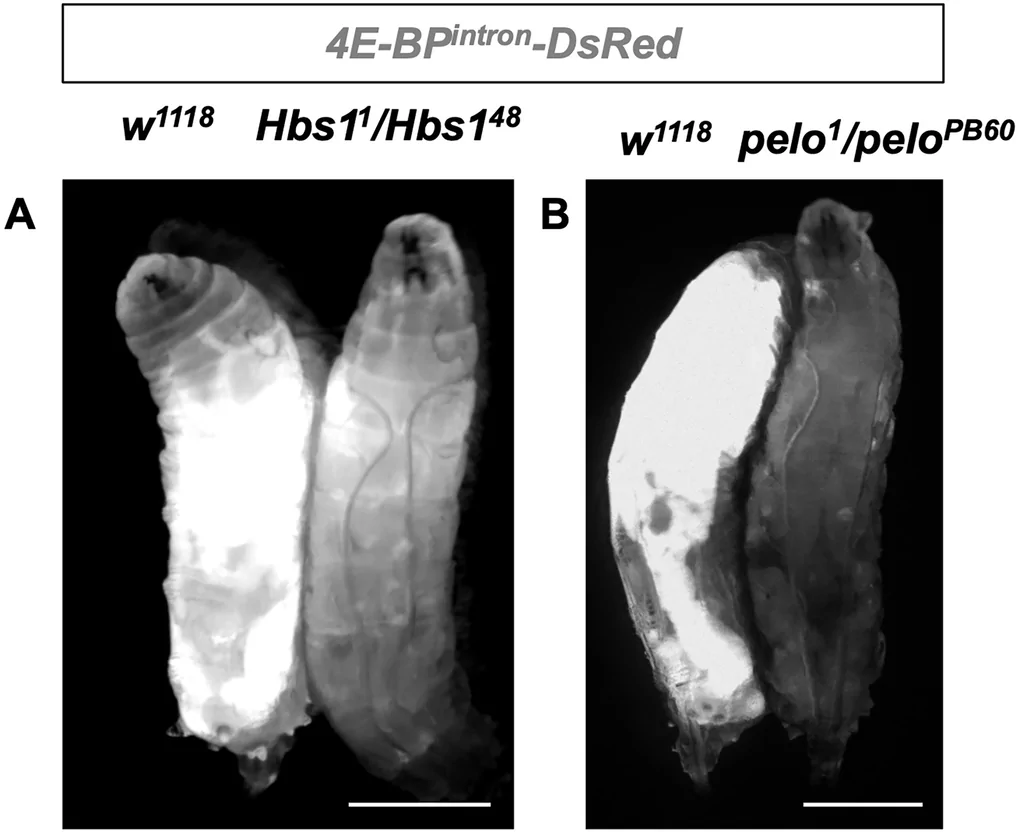
Loss of *Hbs1* or *pelo* results in reduced ATF4 reporter expression. Fluorescence images showing intensity of the ATF4 reporter, 4E-BP^intron^-DsRed in live wandering third instar larva from wild type (*w*^*1118*^), *Hbs1* (**A**) and *pelo* (**B**) mutants. Scale bars represent 1 μm.

To study the effects of the above described release factors on ATF4, we employed a faithful reporter of ATF4 transcriptional activity, 4E-BP^intron^-DsRed, which is constitutively expressed in the wandering third instar larval fat tissue (also known as fat body) (Kang et al, 2016). In the wandering third-instar larval stage, the fat body forms a large, sheet-like organ composed of polyploid adipocyte-like cells that line the inner body cavity beneath the cuticle. We identified the *Drosophila* homologs of all release factor GTPases (Marygold et al, 2016) (Table 1) and utilized the GAL4-UAS system (Brand and Perrimon, 1993) to RNAi deplete them in the fat body. Using a fat body driver, *Dcg-GAL4* (Asha et al, 2003), we found that two independent *UAS-RNAi* lines targeting *Hbs1*, the *Drosophila* homolog of HBS1L, led to reduced ATF4 activity in adipocytes in comparison to adipocytes expressing a control *UAS-LacZ*^*RNAi*^ (Fig. 1A,B). We did not recover any *Dcg* > *eRF3*^*RNAi*^ animals, and we also did not record appreciable changes in DsRed levels with the other RNAi lines tested. Notably, depleting *Hbs1* in the fat body did not influence the expression of a *UAS-GFP* transgene (Fig. 1A), indicating that the effects of Hbs1 were specific to ATF4. We validated these results in *Hbs1* loss of function mutant animals (transheterozygous *Hbs1*^*1*^*/Hbs1*^*48*^ (Li et al, 2019)), which also showed a marked decrease in 4E-BP^intron^-DsRed in the fat body in comparison to wild-type *w*^*1118*^ animals (Figs. 1C,D and EV1A).

**Table 1:** Human and corresponding *Drosophila* orthologs of proteins encoding termination factors tested in the *Drosophila* RNAi screen.

Human	*Drosophila*
eRF3a	Elf, eRF3 (FBgn0020443)
eRF3b	No ortholog known
HBS1L	Hbs1 (FBgn0042712)
GTPBP1	Gtpbp1 (FBgn0027836)
GTPBP2	Gtpbp2 (FBgn0037391)
eRF1	eRF1 (FBgn0036974)
Pelota (Pelo)	pelota (FBgn0011207)

Where available, multiple RNAi lines per gene were used (see Table 2).

Next, we sought to identify whether Pelo, the release factor known to partner with Hbs1, is required for ATF4 activity in the fat body. Fat bodies from *pelo* loss of function mutant animals (transheterozygous *pelo*^*1*^*/pelo*^*PB60*^ (Yang et al, 2015) showed a marked decrease in 4E-BP^intron^-DsRed (Figs. 1E,F and EV1B), similar to what we observed with loss of *Hbs1*. We also sought to test the other known release factor, eRF1. However, *Drosophila eRF1* loss of function mutants have been reported to be lethal (Chao et al, 2003) and we were also unable to recover animals when we knocked down *eRF1* in the fat body. Based on these results, we concluded that the Hbs1 acts together with Pelo to regulate ATF4 in *Drosophila*, and we proceeded to investigate whether such regulation was conserved in vertebrates.

### HBS1L-Pelo regulate ATF4 translation by promoting efficient termination at the upstream ORF

**Figure 2:**
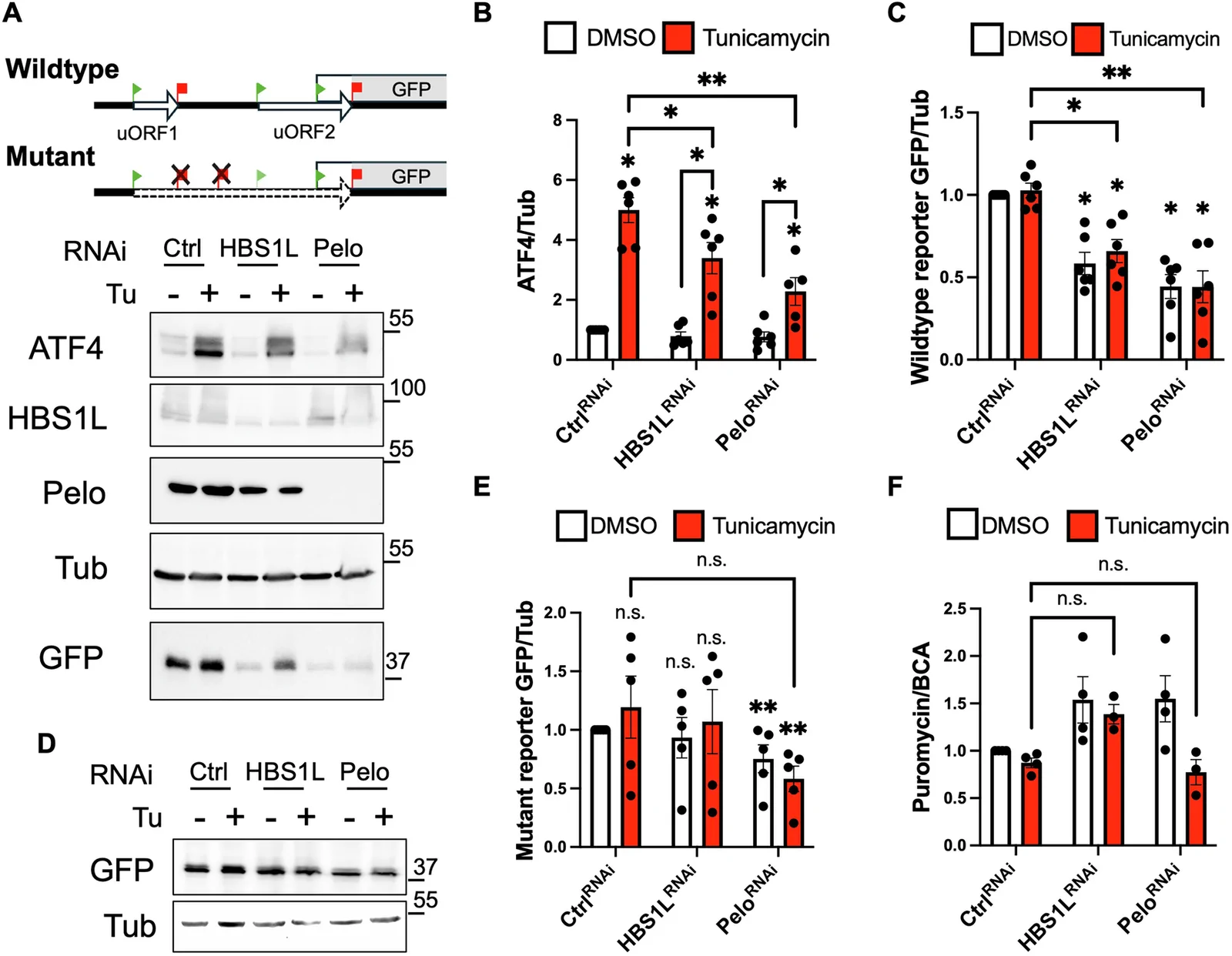
HBS1L and Pelo regulate ATF4 ORF translation by promoting efficient termination. (**A**) (Top) schematic of the wild-type ATF4 5’ leader-GFP reporter and a mutant reporter where all the stop codons upstream of the ATF4 start codon are deleted. Green triangles mark start codons, red squares mark stop codons. Arrows in solid black outline represent open reading frames corresponding to uORFs. Dotted black arrow (bottom) represents the new putative reading frame generated by mutating stop codons in the 5’ leader. (Bottom) Representative western blot analyses of lysates prepared from HEK293T cells co-transfected with siRNA (Ctrl (negative control), HBS1L, Pelo) and a wild-type ATF4 5’ leader-GFP reporter where the coding sequence of ATF4 is replaced with GFP. Cells were treated with 10 μg/ml Tunicamycin (Tu) for 4 h to induce ER stress prior to lysis. Tubulin (Tub) serves as a loading control. (**B**, **C**) Quantification of ATF4 intensity (**B**) and GFP intensity (**C**) from (**A**) normalized to loading control (Tub). Data represent mean of at least five biological replicates; error bars are standard error of mean. *p*-values are as follows: (**B**) columns 1 vs 2 = 0.006; 1 vs 4 = 0.031;1 vs 6 = 0.031; 2 vs 4 = 0.032; 2 vs 6 = 0.002; 3 vs 4 = 0.031; 5 vs 6 = 0.031. (**C**) columns 1 vs 3 = 0.015; 1 vs 4 = 0.015; 1 vs 5 = 0.015; 1 vs 6 = 0.015; 2 vs 4 = 0.032; 2 vs 6 = 0.003. (**D**) Western blot analysis of mutant ATF4 5’ leader-GFP in HEK293T cells where HBS1L and Pelo are RNAi depleted. (**E**) Quantification of GFP signal from (**D**) normalized to Tub. Data represent mean of at least five biological replicates; error bars are standard error of mean. *p*-values are as follows: columns 1 vs 5 = 0.007; 1 vs 6 = 0.007. (**F**) Quantification of Puromycin signal normalized to total protein (as measured by BCA) in HEK293T cells where HBS1L and Pelo are RNAi depleted. Data represent mean of four biological replicates; error bars are standard error of mean. See EV3A for representative blot. Data information: Statistical comparisons with Ctrl^RNAi^-DMSO were performed using the Wilcoxon signed rank test for and are shown as floating asterisks when significant. Comparisons with Ctrl^RNAi^-Tunicamycin were performed using the Friedman test with Dunn’s correction. Other comparisons were performed using the Wilcoxon matched pairs signed rank test. **p < 0.01, *p < 0.05, n.s. = not significant. Source data are available online for this figure.

**Figure EV2:**
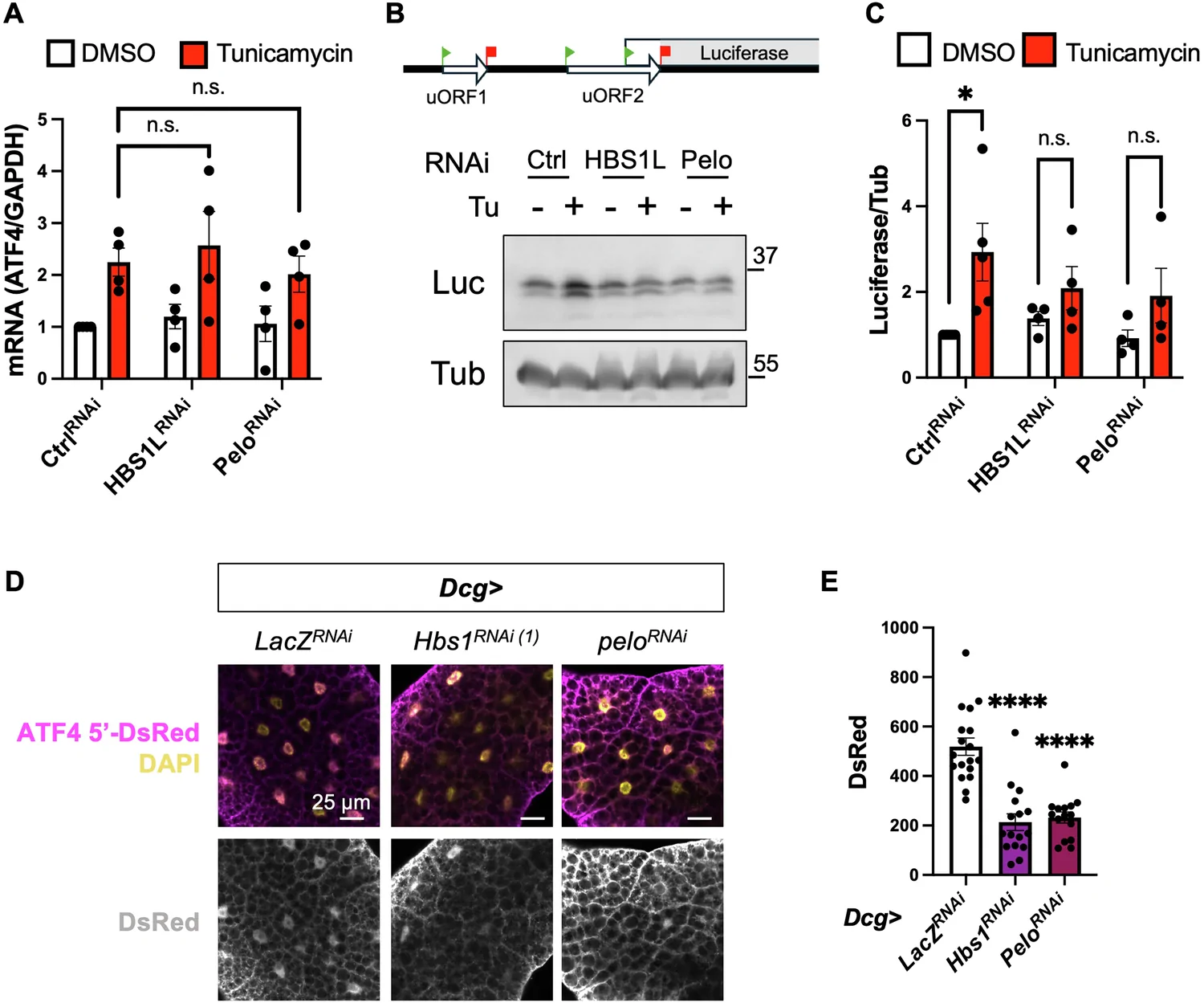
HBS1L and Pelo act on the 5’ leader of human and *Drosophila* ATF4. (**A**) q-RTPCR data measuring ATF4 mRNA normalized to house-keeping gene (GAPDH) in cells transfected with siRNA targeting HBS1L or Pelo and treated with 10μg/ml Tunicamycin to induce ER stress. Data represent mean of four biological replicates; error bars are standard error of mean. Statistical comparisons with Ctrl^RNAi^-DMSO were performed using the Wilcoxon signed rank test for and are shown as floating asterisks when significant. Comparisons with Ctrl^RNAi^-Tunicamycin were performed using the Friedman test with Dunn’s correction. Other comparisons were performed using the Wilcoxon matched pairs signed rank test. (**B**) (Top) Schematic of the wild-type ATF4 5’ luciferase reporter and (bottom) reporter expression as measured by western blotting in cells treated as indicated. (**C**) Quantification of luciferase signal in (**B**) normalized to loading control (Tubulin). Data represent mean of at least four biological replicates; error bars are standard error of mean. Statistical comparisons were made using the Mann–Whitney test. *p*-values are as follows: columns 1 vs 2 = 0.03. (**D**) Representative confocal images of fat bodies expressing an ATF4 5’leader-DsRed reporter (magenta) in animals where *Hbs1* or *pelo* are depleted using *Dcg-GAL4*. DAPI marks nuclei in yellow. (**E**) Quantification of DsRed intensity from (**D**). Each data point represents a region of interest from a single animal, black bar represents mean of data from at least 5–7 biological replicates. Error bars represent standard error of mean. Statistical comparisons were made using Kruskal–Wallis test with Dunn’s correction. *p*-values are approximate. Data information: ****p < 0.0001, ***p < 0.001, **p < 0.01, *p < 0.05, n.s. = not significant.

To test whether HBS1L-Pelo regulate ATF4 in human cells, we siRNA-depleted these factors in HEK293T cells. To robustly detect ATF4, we treated cells with the ER stress-inducing chemical, Tunicamycin. Western blot analyses revealed that the siRNA-depletion indeed led to reduced HBS1L and Pelo protein in cells (Fig. 2A). While cells transfected with control siRNA showed nearly five-fold induction in ATF4 with ER stress, depleting either HBS1L or Pelo resulted in significantly lower ATF4 levels (Fig. 2A,B). These results strongly suggested that HBS1L- and Pelo-mediated ATF4 regulation is conserved between *Drosophila* and humans. Further, we observed no change in ATF4 mRNA levels in HEK293T cells with siRNA treatment (Fig. EV2A), leading us to posit that HBS1L-Pelo likely regulate ATF4 translation.

ATF4 translation is heavily regulated via the 5’ leader of the ATF4 mRNA. To test whether HBS1L and Pelo act on the 5’ leader of ATF4 mRNA, we utilized a reporter where the wild-type ATF4 5’ leader is placed upstream of GFP (Lu et al, 2004) (schematic, Fig. 2A). Consistent with our hypothesis, we observed that knockdown of either HBS1L or Pelo resulted in reduced ATF4 5’-GFP reporter levels under both vehicle-treated and ER stress conditions (Fig. 2A,C). We’d like to note here that consistent with previous reports, the ATF4 5’-GFP reporter does not show appreciable inducibility with ER stress unlike endogenous ATF4 even in cells transfected with control siRNA. We attribute to this to the perdurance of GFP which is proteostatically more stable in comparison to endogenous ATF4 (Lu et al, 2004). To test this, we also utilized a luciferase-based reporter, where the wild-type ATF4 5’ leader is placed upstream of renilla luciferase(Bohlen et al, 2020). We observed that knockdown of HBS1L or Pelo led to reduced luciferase activity in comparison to control siRNA cells (Fig. EV2B,C). We further validated these results using a *Drosophila UAS-ATF4 5’leader-DsRed* reporter(Walsh et al, 2025), where we observed that knocking down of either *Hbs1* or *pelo* resulted in reduced DsRed levels (Fig. EV2D,E). Together, these results conclusively show that HBS1L-Pelo regulate translation of ATF4 via the 5’ leader region.

**Figure EV3:**
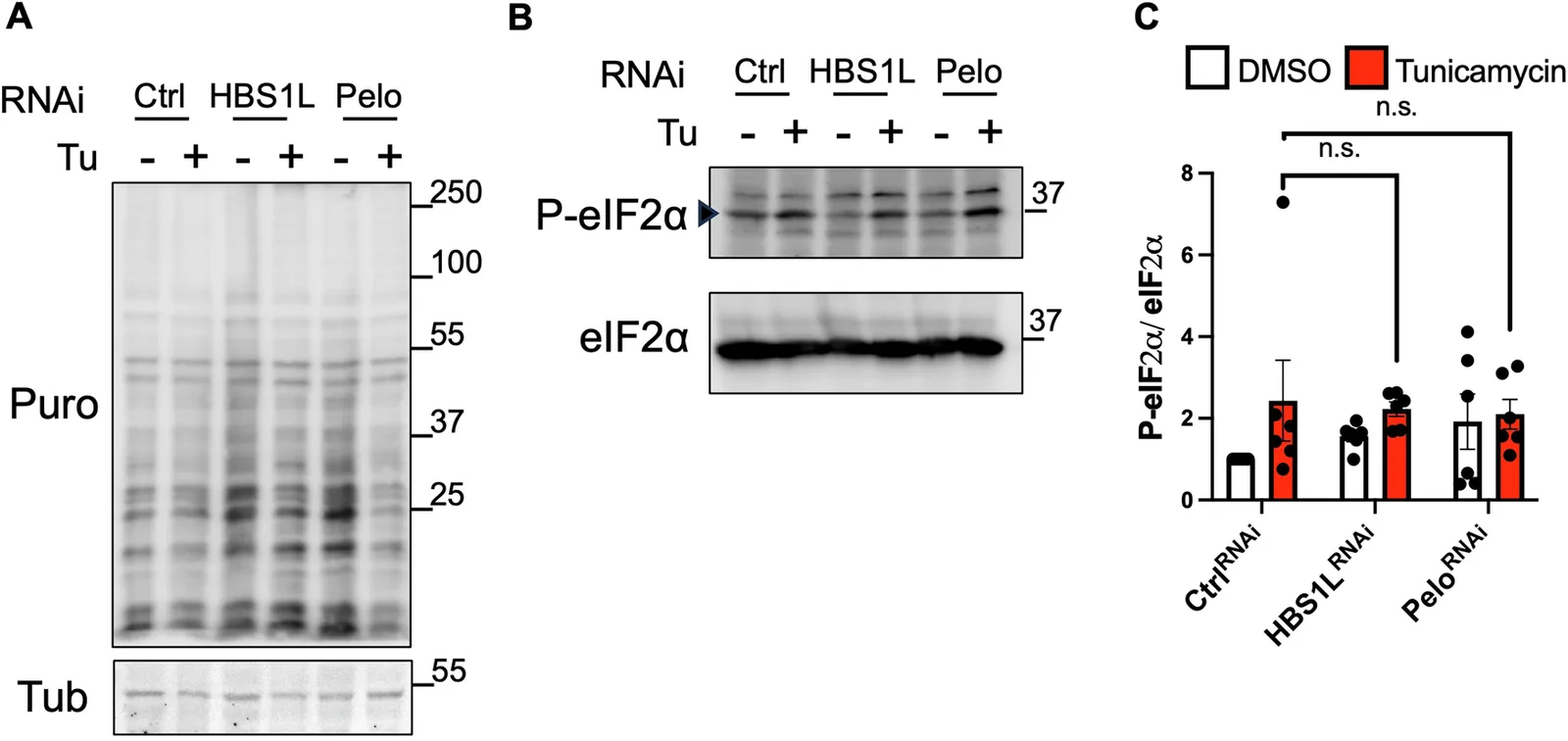
Depleting HBS1L or Pelo does not have an effect on global translation or phosphorylation of eIF2α. (**A**) Representative western blot for puromycin-incorporation in cells where HBS1L or Pelo is depleted. Quantification presented in Fig. 2F. (**B**) Western blot analysis of phospho-eIF2α (P-eIF2α) and total eIF2α in cells treated as indicated. Black arrowhead in upper panel indicates relevant P-eIF2α band. (**C**) Quantification of P-eIF2α from (**B**) normalized to total eIF2α. Data represent mean of at least five biological replicates; error bars are standard error of mean. Statistical comparisons were made using Kruskal–Wallis test with Dunn’s correction. n.s. = not significant.

Since the best studied role for HBS1L-Pelo is in ribosome recycling (Atkinson et al, 2008; Pisareva et al, 2011), we further hypothesized that HBS1L-Pelo likely facilitate reinitiation at the ATF4 main ORF by promoting efficient termination at stop codons preceding the ATF4 start codon. Such efficient termination would allow for DENR-MCTS1/eIF2D to effectively remove spent tRNAs and generate reinitiation-competent 40S ribosomes. If this model is correct, eliminating all termination events preceding reinitiation at the ATF4 start codon should eliminate the effects of HBS1L-Pelo on ATF4 translation. To test this, we used a mutant version of the ATF4 5’-GFP reporter wherein all stop codons in the 5’ leader are mutated (Lu et al, 2004) (schematic, Fig. 2A). Consistent with our model, we observed that siRNA knockdown of HBS1L did not impact levels of the mutant reporter (Fig. 2D,E). Interestingly, knockdown of Pelo resulted in a small but statistically significant reduction in the mutant reporter. This could be because loss of Pelo results in a global decrease in translation, which we tested using a puromycin incorporation assay to measure global translation rates in HBS1L and Pelo knockdown cells. Our data showed that depleting either HBS1L or Pelo did not result in a statistically significant change in overall translation rates under vehicle-treated or ER stress conditions (Figs. 2F and EV3A) and also did not impact phosphorylation of eIF2α (Fig. EV3B,C). However, we did observe that the relative decrease in translation rates between vehicle-treated and ER stress conditions was much more pronounced in Pelo siRNA cells in comparison to control siRNA cells (Figs. 2F and EV3B). This analysis suggests that the decrease in ATF4 protein seen with Pelo depletion in Fig. 2A,B may be partly due to a decrease in global translation seen with loss of Pelo. Nonetheless, our combined results support a model wherein HBS1L-Pelo mediate efficient translation termination at uORF1 and thus promote reinitiation at the ATF4 ORF.

### Hbs1 mutants exhibit phototransduction defects

Our data thus far showed Hbs1 and Pelo to be regulators of ATF4 in both *Drosophila* and human cells. Human patients with loss-of-function mutations in *HBS1L* display several developmental defects, including facial dysmorphia, restricted growth, and hyperpigmented deposits in their retina, and phototransduction defects as measured by electroretinography (ERG) (O’Connell et al, 2019; Luo et al, 2024). Recent work with mouse models has revealed that *HBS1L* deletion results in the loss of several retinal neurons, which likely underlie the vision defects seen in human patients (Luo et al, 2024). However, it remains unknown which cell types in the visual system rely on HBS1L for their proper development and function. Further, specific HBS1L mRNA targets that cause vision defects have not been described. To address these open questions, we utilized *Drosophila Hbs1* mutants to model the vision defects observed in HBS1L deficiency patients.

**Figure 3:**
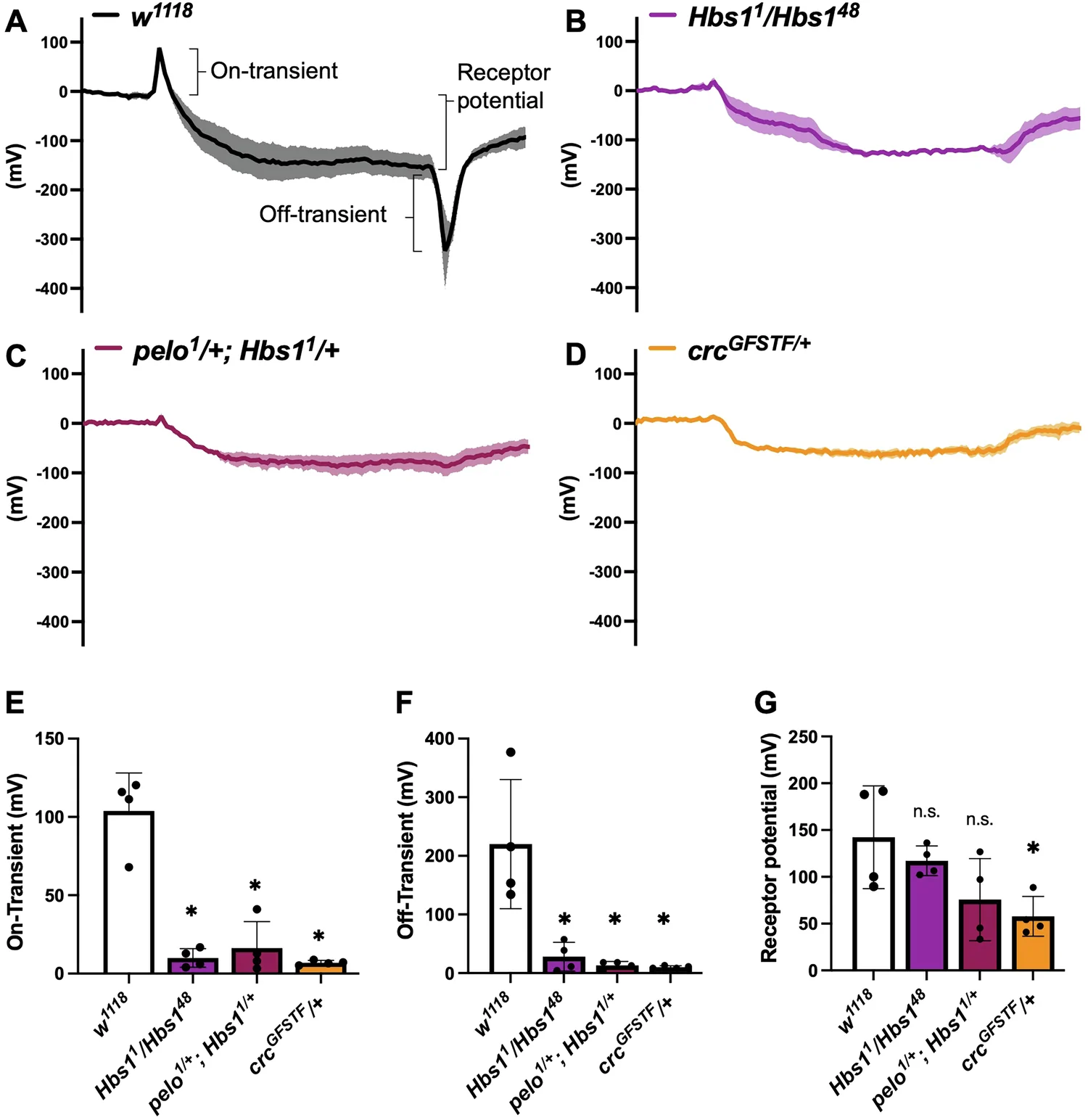
Loss of *Hbs1, pelo*, or *ATF4* results in phototransduction defects. (**A**–**D**) Representative ERG traces from 3–5-day-old wild type (*w*^*1118*^, **A**), *Hbs1* mutants (*Hbs1*^*1*^*/Hbs1*^*48*^, **B**), *Hbs1* and *pelo* hemizygous mutants (*pelo*^*1*^*/+;Hbs1*^*48*^*/+*, **C**), and *ATF4* heterozygous mutants (*crc*^*GFSTF*^*/+*, **D**). The solid line represents average of three technical replicates, and shaded error envelope represents the standard error of mean across the measurements. The three quantifiable parameters of ERGs, on-transient, off-transient, and receptor potential are marked by parentheses in (**A**). (**E**–**G**) Quantification of on-transient (**E**), off-transient (**F**), and receptor potential (**G**) from (**A**–**D**). Data bars are color coded to genotypes in (**A**–**D**). Each data point is the average of three technical replicates. Data information: Datasets represent the mean from at least four biological replicates recorded on separate days; error bars are standard error of mean. Statistical comparisons were made using the Kruskal–Wallis test with Dunn’s correction. *p*-values are as follows: (**E**) 1 vs 2 = 0.028; 1 vs 3 = 0.028; 1 vs 4 = 0.028. (**F**) 1 vs 2 = 0.028; 1 vs 3 = 0.028; 1 vs 4 = 0.028. (**G**) 1 vs 4 = 0.028. *p < 0.05, n.s. = not significant. Source data are available online for this figure.

We first used ERG measurements to examine whether *Hbs1* mutant animals showed vision defects in comparison to the control wild-type *w*^*1118*^ animals. The compound eye in *Drosophila* is composed of ~800 individual unit eyes called ommatidia, each containing eight photoreceptors (Sanes and Zipursky, 2010). Outer photoreceptors (R1–R6) in the retina project to the first optic neuropil, the lamina, where they synapse with lamina neurons in modular cartridges. A typical ERG trace in control animals contains an initial sustained receptor potential from photoreceptor depolarization, as well as “on” and “off” transients at light onset and offset that reflect synaptic transmission to downstream lamina neurons (*Drosophila* equivalent of bipolar cells) (Heisenberg, 1971; Alawi and Pak, 1971; Coombe and Heisenberg, 1986; Rhodes-Mordov et al, 2015) (Fig. 3A). Since ERG analyses are known to be sensitive to eye pigmentation (Stark, 1973), we ensured that all our comparative analyses were across animals that had similar genetic expression of pigment genes.

**Figure EV4:**
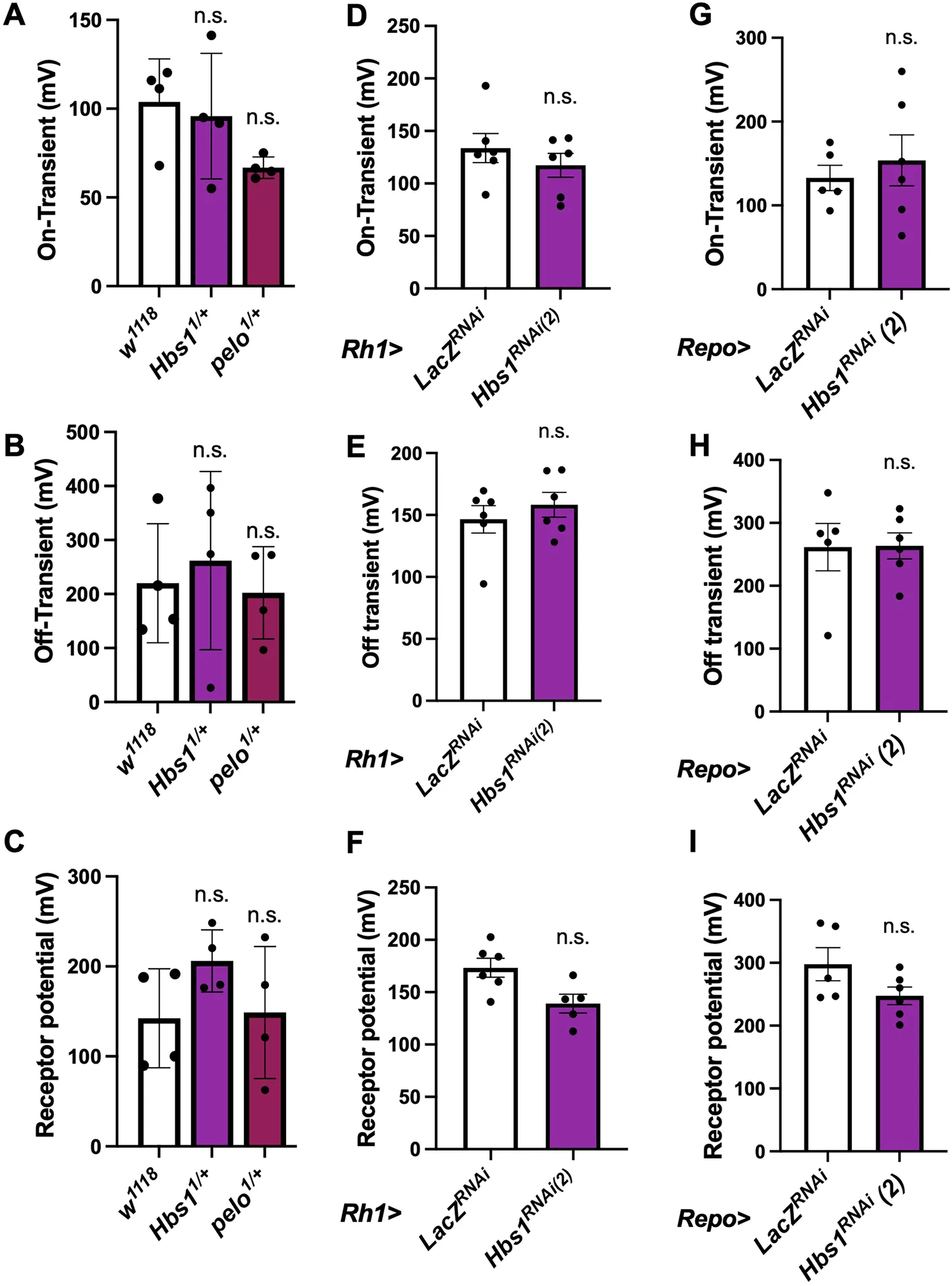
Depleting *Hbs1* in photoreceptors or glia does not impact phototransduction. (**A**–**C**) Quantification of on-transient (**A**), off-transient (**B**), and receptor potential (**C**) from 3–5-day-old animals from control (*w*^*1118*^), *Hbs1* heterozygous (*Hbs1*^*1*^*/+*) and *pelo* heterozygous (*pelo*^*1*^*/+*). Each data point is the average of three technical replicates. Datasets represent the mean from at least four biological replicates; error bars are standard error of mean. Please note that the control data set here is the same as in Fig. 3 and has been replotted for convenience. Statistical comparisons were made using the Kruskal–Wallis test with Dunn’s correction. (**D**–**I**) Quantification of on-transient (**D**, **G**), off-transient (E, H), and receptor potential (**F**, **I**) from 3–5-day-old animals where either a photoreceptor driver (*Rh1-*GAL4) or a glia driver (*Repo-GAL4*) is used to express a control *UAS-LacZ*^*RNAi*^ or -*Hbs1*^*RNAi*^. Each data point is the average of three technical replicates. Data information: Datasets represent the mean from at least four biological replicates; error bars are standard error of mean. Statistical comparisons were made using the Mann–Whitney test. n.s. = not significant.

In young (3–5 day old) animals, we found that loss of *Hbs1* resulted in a blunted ERG response to a single light stimulus (Fig. 3A,B). Quantification of the ERG parameters showed that *Hbs1* mutants had a significant decrease in on- and off-transients in comparison to control *w*^*1118*^ animals (Fig. 3E,F). These data suggested that the vision defects in *Hbs1* mutants may be due to either improper synaptic transmission between the photoreceptor and lamina layers or due to defective lamina function. We also observed a small but statistically insignificant decrease in receptor potential in mutant animals, indicating that photoreceptor depolarization was largely unaffected (Fig. 3G). We observed similar results with animals hemizygous for both *Hbs1* and *pelo* (Fig. 3C,E–G), suggesting that Hbs1 acts as a complex with Pelo in the context of visual system function. We note here that we were unable to recover homozygous or transheterozygous *pelo*^*1*^ *or pelo*^*PB60*^ adults isogenized to the *w*^*1118*^ background and hence opted to test animals hemizygous for *Hbs1* and *pelo*. Further, we also did not observe any ERG defects with *Hbs1* heterozygous mutants or *pelo* heterozygous mutants (Fig. EV4A–C), suggesting that combined loss of Hbs1 and Pelo drive the ERG defects seen in Fig. 3C,E–G.

Finally, given our results that ATF4 is regulated by Hbs1-Pelo in the fat body (Fig. 1), we asked whether loss of ATF4 phenocopies ERG defects seen in *Hbs1* mutants. Since ATF4 (encoded by *cryptocephal, crc* in *Drosophila)* null mutants do not survive to adulthood, we used a hypomorphic allele of ATF4 (*crc*^*GFSTF*^*/+*) (Vasudevan et al, 2022). Consistent with our previous report that loss of ATF4 results in age-dependent retinal degeneration, *crc*^*GFSTF*^*/+* animals showed blunted ERG traces and reduced on- and off-transients similar to *Hbs1* and *pelo* mutant animals (Fig. 3D–F). However, loss of ATF4 also resulted in reduced receptor potential, indicating that these animals may also have defective photoreceptor function in addition to lamina defects (Fig. 3G). Nonetheless, these phenotypic analyses demonstrate that loss of Hbs1, its binding partner Pelo, and its mRNA target *ATF4* all result in strikingly similar phototransduction defects.

### Hbs1-Pelo is required in the lamina layer for proper phototransduction

**Figure 4:**
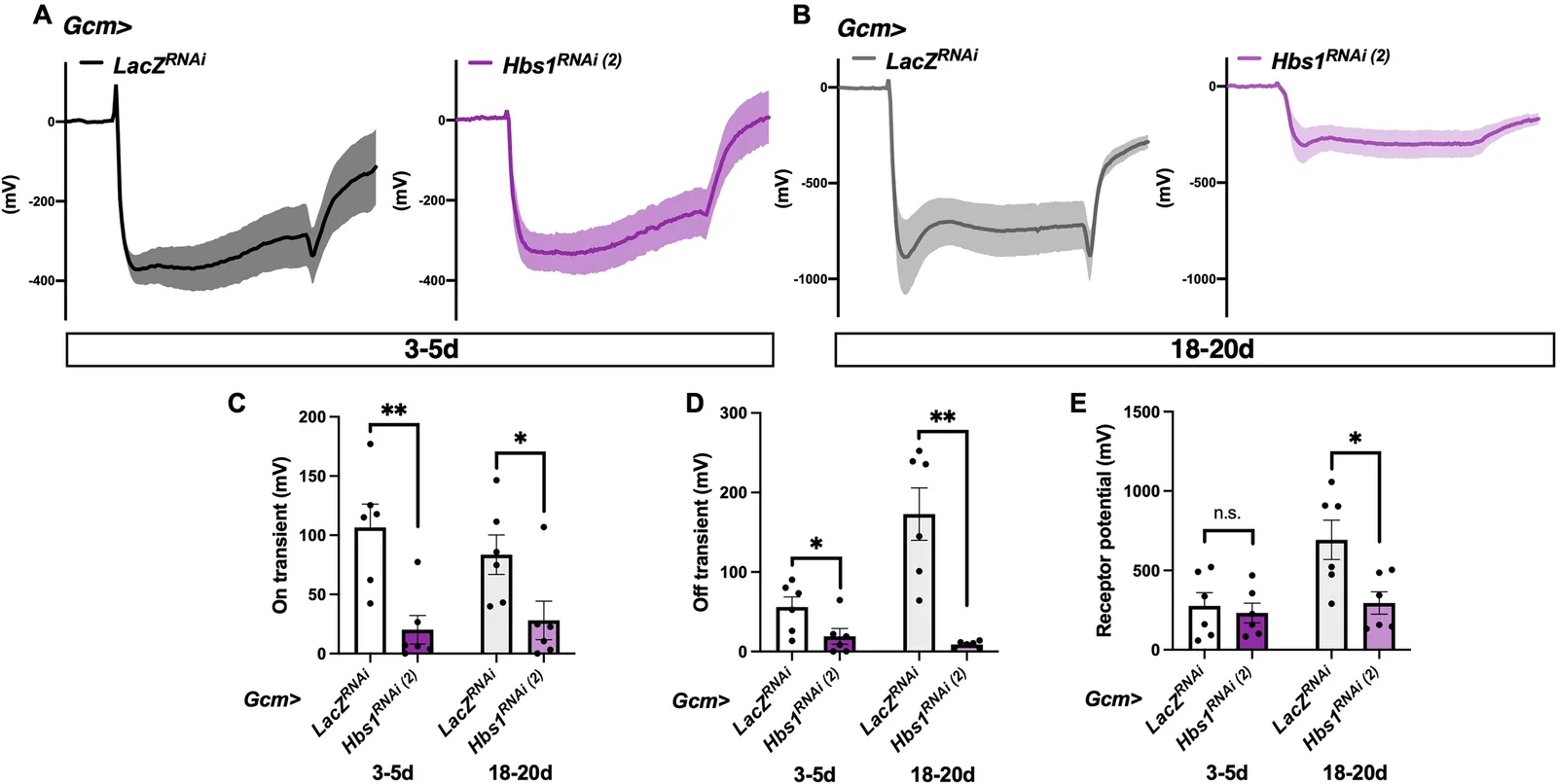
Depleting *Hbs1* in the lamina recapitulates phototransduction defects seen in *Hbs1* mutants. (**A**,** B**) Representative ERG traces from 3–5-day-old (**A**) or 18–20-day-old (**B**) animals where a lamina driver (*Gcm-GAL4*) is used to deplete *Hbs1*. Data are graphed as in Fig. 3A–D. (**C**–**E**) Quantification of on-transient (**C**), off-transient (**D**), and receptor potential (**E**) from animals in (**A**, **B**). Data are plotted as in Fig. 3E–G. Statistical comparisons were made using the Mann–Whitney test. *p*-values are as follows: (**C**) 1 vs 2 = 0.008; 3 vs 4 = 0.026. (**D**) 1 vs 2 = 0.041; 3 vs 4 = 0.002. (**E**) 3 vs 4 = 0.041. Data information: **p < 0.01, *p < 0.05, n.s. = not significant. Source data are available online for this figure.

We next sought to use the *Drosophila* genetic toolkit to determine which cell types in the visual system rely on Hbs1-Pelo for proper function. To do so, we used cell type-specific drivers to RNAi deplete *Hbs1* throughout development and subjected young adult animals to ERG analysis. *Hbs1*, *pelo*, and *ATF4* mutants show significant loss in on- and off-transients, which are indicative of lamina defects (Fig. 3). We asked whether depleting *Hbs1* in either photoreceptors (using *Rh1-GAL4* (Yoshihara et al, 1999; Hall et al, 2017)) or in the lamina (using *Gcm-GAL4* (Jenett et al, 2012; Chen et al, 2016)) during development was sufficient to recapitulate the ERG defects in *Hbs1* mutants. We observed no significant ERG defects in *Rh1>Hbs1*^*RNAi*^ animals (Fig. EV4D–F). However, 3–5-day-old *Gcm>Hbs1*^*RNAi*^ animals showed reduced on- and off-transients with no change to receptor potential (Fig. 4A,C–E), similar to the ERG profiles we observed for *Hbs1* mutants.

The lamina contains five main classes of neurons (L1–L5) that receive direct synaptic input from the outer photoreceptor terminals (R1–R6) (Sanes and Zipursky, 2010). It also houses several types of glial cells—epithelial, marginal, and satellite glia—that support lamina neuron function and metabolism. Notably, the *Gcm-GAL4* driver is expressed in epithelial and marginal glial populations and in the lamina neurons. We used a pan-glial driver, *Repo-GAL4* (Sepp et al, 2001), to test whether Hbs1 was required in the lamina glial populations. ERG analysis revealed no significant differences in the ERG parameters between control *Repo>LacZ*^*RNAi*^ and *Repo>Hbs1*^*RNAi*^ animals (Fig. EV3), suggesting that Hbs1 is not required in lamina glia. This data led us to consider that Hbs1 is likely required in lamina neurons.

**Figure 5:**
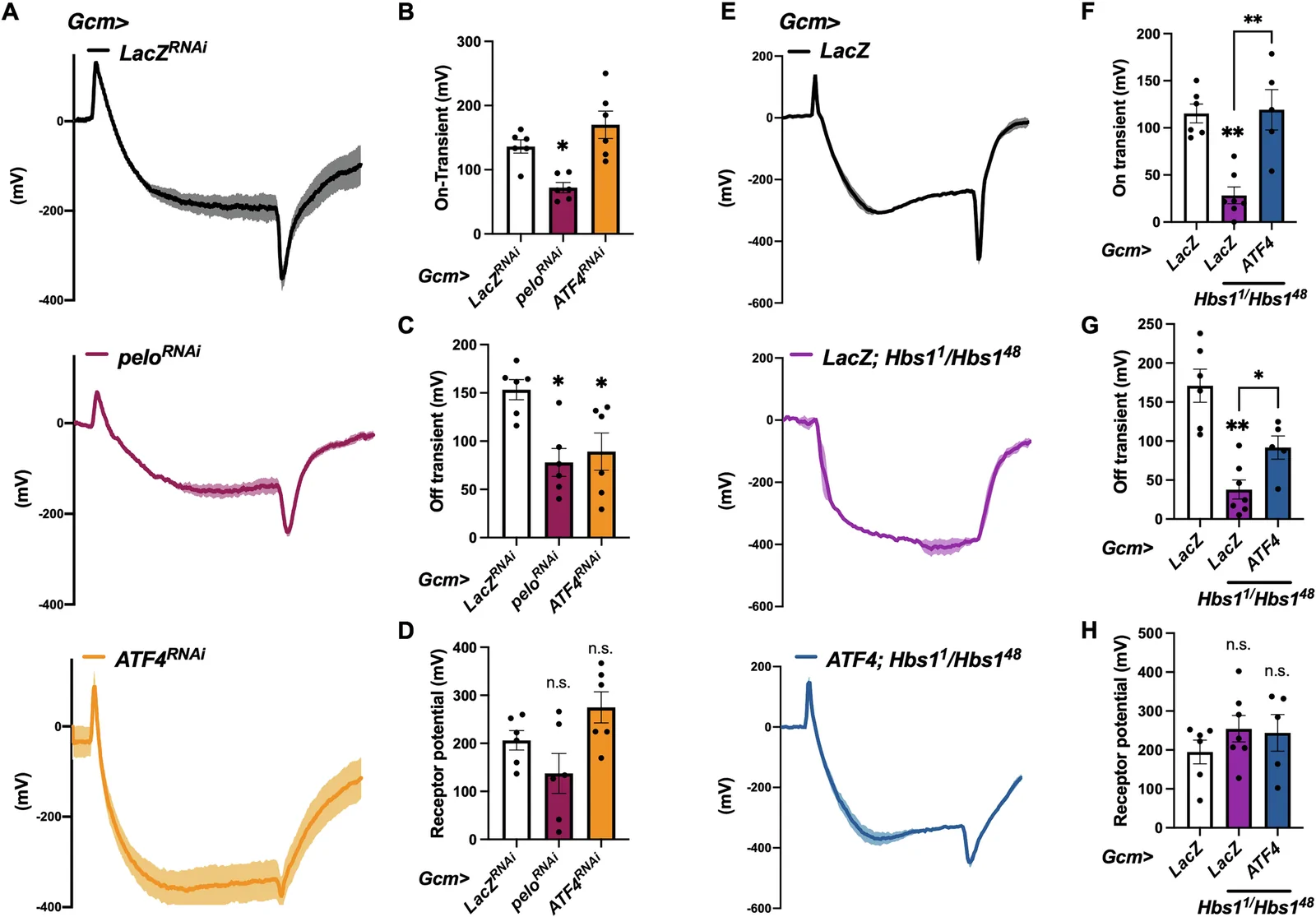
Restoring ATF4 expression in the lamina partially rescues ERG defects in *Hbs1* mutants. (**A**) Representative ERG traces from 3–5-day-old animals where *Gcm-GAL4* is used to deplete *pelo* (middle, maroon trace), *ATF4* (bottom, orange trace) or express a control *LacZ*^*RNAi*^ (top, black trace). Data are graphed as in Fig. 3A–D. (**B**–**D**) Quantification of on-transient (**B**), off-transient (**C**), and receptor potential (**D**) from animals in (**A**). Data are plotted as in Fig. 3E–G. Statistical comparisons were made using Kruskal–Wallis test with Dunn’s correction. *p*-values are as follows: (**B**) columns 1 vs 2 = 0.029. (**C**) columns 1 vs 2 = 0.025; 1 vs 3 = 0.017. (**E**) Representative ERG traces from 3–5-day-old animals where *Gcm-GAL4* drives expression of a control *UAS-LacZ* transgene in wild-type animals (top, black trace), *Hbs1* mutants (middle, purple trace) or of a leaderless *ATF4* in *Hbs1* mutants (bottom, blue trace). Data are graphed as in Fig. 3A–D. (**F**–**H**) Quantification of on-transient (**F**), off-transient (**G**), and receptor potential (**H**) from animals in (**A**). Data are plotted as in Fig. 3E–G. Statistical comparisons with *Gcm>LacZ* were made using the Kruskal–Wallis test with Dunn’s correction and are indicated as floating asterisks when significant. Other comparisons were made using the Mann–Whitney test. *p*-values are as follows: (**F**) columns 1 vs 2 = 0.006; 2 vs 3 = 0.005. (**G**) 1 vs 2 = 0.001; 2 vs 3 = 0.048. Data information: **p < 0.01, *p < 0.05, n.s. = not significant. Source data are available online for this figure.

Previous studies have demonstrated that defects in lamina neurons can result in an age-related loss in photoreceptor function (Soukup et al, 2013; Lee and Sun, 2015). Based on this, we considered whether old *Gcm>Hbs1*^*RNAi*^ animals may exhibit receptor potential defects in ERG analyses, in addition to on- and off-transient defects seen in young animals. Indeed, we found that in comparison to age-matched control *Gcm>LacZ*^*RNAi*^ animals, 18–20 day *Gcm>Hbs1*^*RNAi*^ animals show a marked decrease in their receptor potential, which is reflective of photoreceptor defects (Fig. 4B,E). Additionally, we also observed a larger decrease in on- and off-transients in 18–20 day *Gcm>Hbs1*^*RNAi*^ animals when compared to 3–5 day animals (Fig. 4C,D), suggestive of further age-related impairment of lamina neuron function as well. Given than *Gcm>Hbs1*^*RNAi*^ produced defects in on- and off-transients in young adults, these data suggest that defects in lamina neuron function may be acquired during development. We also recorded similar ERG defects in *Gcm>pelo*^*RNAi*^ animals (Fig. 5A–D), suggesting that Hbs1 acts together with Pelo in the visual system. This led us to propose a model where Hbs1-Pelo is required for lamina neuron development and function, which can in turn impact photoreceptor function with age.

### Restoring ATF4 in the lamina partially rescues phototransduction defects in *Hbs1* mutants

Though previous translation profiling studies in *HBS1L* deletion patient fibroblasts showed changes in translation efficiency of many mRNAs (O’Connell et al, 2019; Luo et al, 2024), none of them have been linked to the etiology of vision defects observed in patients. Given our findings that HBS1L-Pelo regulates ATF4 (Figs. 1 and 2) and the phenotypic similarities in ERGs from *Hbs1* and *ATF4* mutants (Fig. 3), we asked whether the vision defects with loss of *Hbs1* may partly be due to reduced ATF4 levels. To test this possibility, we first examined whether depleting *ATF4* in the lamina phenocopies phototransduction defects seen with lamina-specific *Hbs1* depletion. ERG measurements from young *Gcm* > *ATF4*^*RNAi*^ animals showed decreased off-transients but with no change to on-transient or photoreceptor potential (Fig. 5A–D), suggesting a possible role for ATF4 in the lamina.

We next asked whether restoring ATF4 protein in the lamina was sufficient to rescue the ERG defects seen in *Hbs1* mutants. To do so, we drove expression of a leaderless *UAS-ATF4* (Vasudevan et al, 2020) or a control *UAS-LacZ* using *Gcm-GAL4* in *Hbs1*^*1*^*/Hbs1*^*48*^ animals. Consistent with our data in *Hbs1* mutants (Fig. 3A,B,E–G), our ERG analyses revealed that animals expressing control *LacZ* (*Gcm>LacZ*) in the *Hbs1*^*1*^*/Hbs1*^*48*^ background resulted in on- and off-transient defects when compared to control *w*^*1118*^ animals (Fig. 5E–H). These defects in *Hbs1*^*1*^*/Hbs1*^*48*^ animals were partially rescued by *Gcm* > *ATF4* expression (Fig. 5E–H), demonstrating that ATF4 expression is sufficient to rescue vision defects seen with loss of *Hbs1*. While these experiments do not conclusively demonstrate that ATF4 is the relevant downstream target of Hbs1 in the lamina, the vision defects in *Gcm* > *ATF4*^*RNAi*^ (Fig. 5A–D) and the rescue experiments (Fig. 5E–H) together suggest that Hbs1-Pelo-mediated regulation of ATF4 is required for proper lamina function.

### *Hbs1* mutants show vacuolation defects in the lamina layer

**Figure 6:**
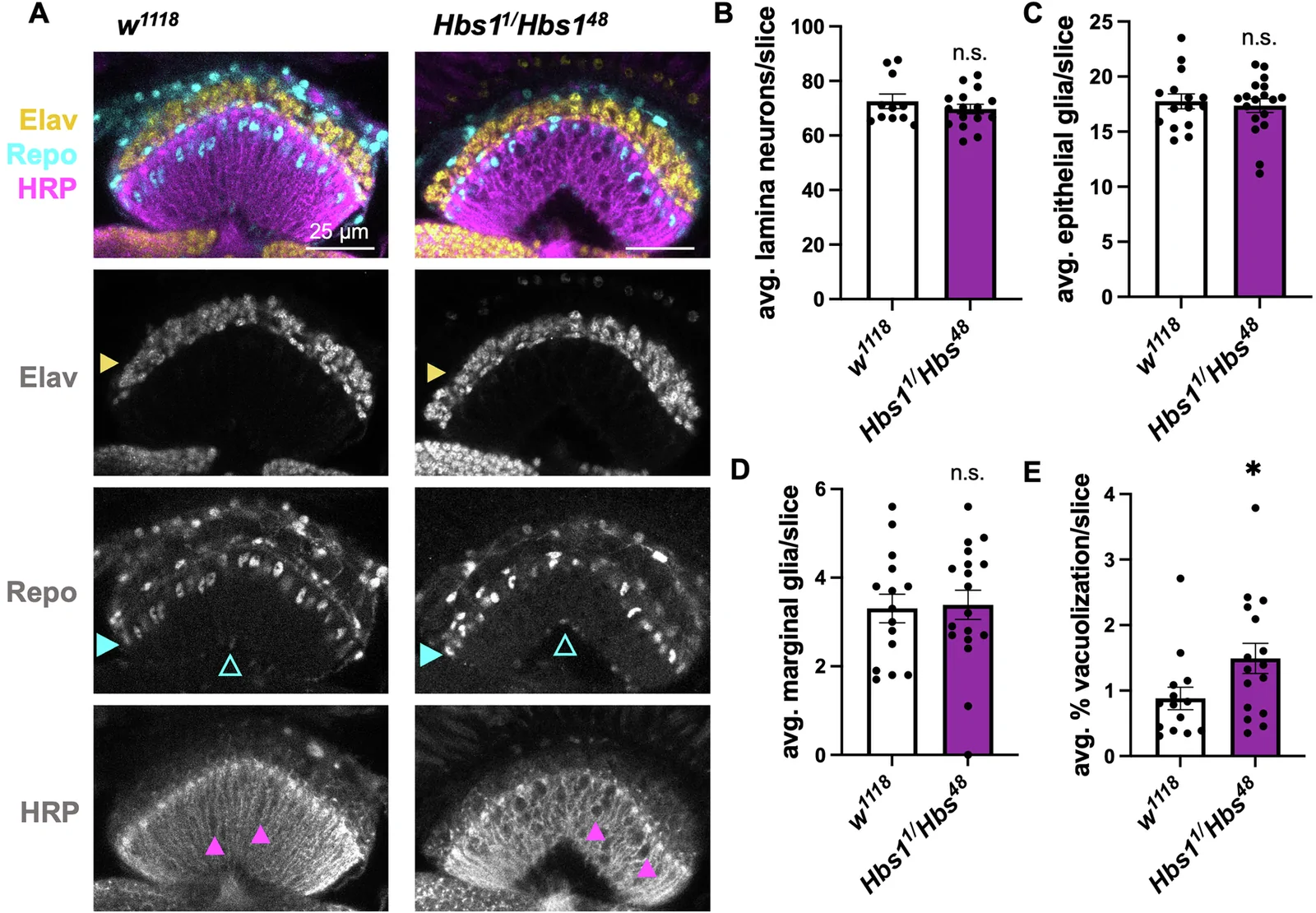
Loss of *Hbs1* results in increased vacuolization in the lamina neuropil with no effect on the number or morphology of lamina neurons and glia. (**A**) Confocal images of 3–5-day-old adult lamina from wild-type or *Hbs1* mutant animals stained with a neuronal soma marker (Elav, yellow), glia soma marker (Repo, cyan), and neuronal membrane marker (HRP, magenta). Yellow solid arrows point to lamina neurons, cyan solid arrows point to epithelial glia, cyan open arrows point to marginal glia, magenta solid arrows point to areas of vacuolization. (**B**) Quantification of the total number of lamina neurons in wild-type and *Hbs1* mutants as calculated by summation of individual lamina neurons subtypes (L1–L5) from Fig. EV5A,B. Each data point represents one animal, data bars represent the mean of at least ten biological replicates, error bars represent standard error of mean. (**C**, **D**) Quantification of the number of epithelial (**C**) and marginal (**D**) glia from (**A**). Epithelial glia are identified as Repo-positive cells distal to the lamina neuropil and marginal glia are Repo-positive cells proximal to the lamina neuropil (see cyan arrows in **A**). Each data point represents one animal, data bars represent the mean of at least ten biological replicates, error bars represent standard error of mean. (**E**) Quantification of the percentage of vacuolization as measured by the area of the vacuoles normalized to the total area of the lamina neuropil. Each data point represents one animal, data bars represent the mean of at least ten biological replicates, error bars represent standard error of mean. *p* = 0.038. Data information: Statistical comparisons were made using the Mann–Whitney test. *p < 0.05, n.s. = not significant. Source data are available online for this figure.

**Figure EV5:**
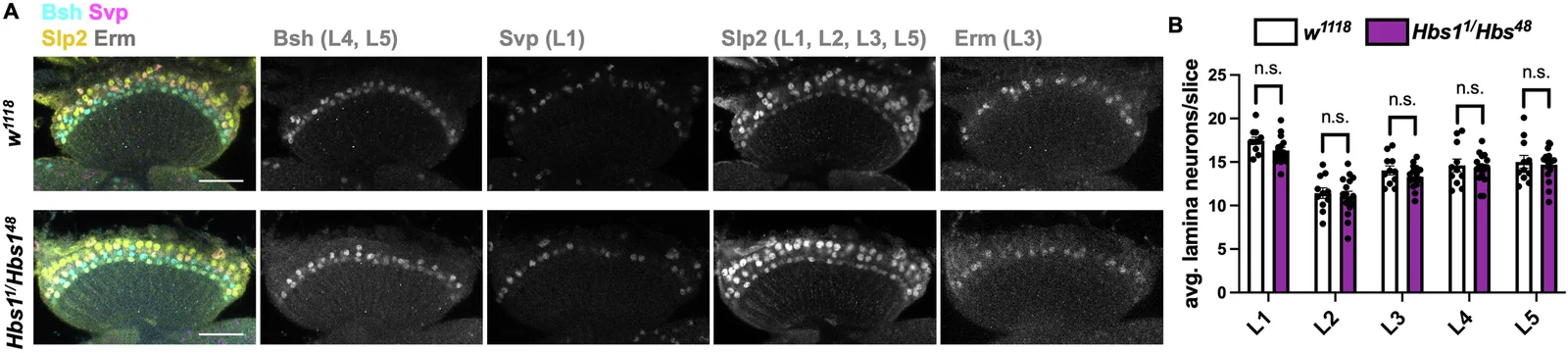
Loss of *Hbs1* does not alter lamina neuron number or morphology. (**A**) Confocal images of 3–5-day-old adult lamina from wild-type or *Hbs1* mutant animals stained markers for L1–L5 lamina neuron subtypes (Slp2 in cyan, Svp in magenta, Erm in yellow, and Bsh in gray). L1 neurons are Svp- and Slp2-positive, L2 neurons are only Slp2-positive, L3 are Erm-positive, L4 are only Bsh-positive, and L5 are Bsh- and Slp2-positive. (**B**) Counts of each lamina neuron subtype as described previously. Each data point represents one animal, data bars represent the mean of at least ten biological replicates, error bars represent standard error of mean. Statistical comparisons were made using the Mann–Whitney test. ****p < 0.0001, n.s. = not significant.

Since our phenotypic analyses with ERG revealed that vision defects in *Hbs1* mutants stem from improper lamina neuron function, we used confocal microscopy to investigate the lamina cortex and neuropil in *Hbs1*^*1*^*/Hbs1*^*48*^ animals. We stained whole eye-optic lobe preparations from *Hbs1* mutants with a pan-neuronal marker Elav (Robinow and White, 1988, 1991). Z-stack analysis of confocal images revealed that loss of *Hbs1* had no obvious impact on organization of the lamina neuron cell bodies in comparison to wild-type animals (Fig. 6A,B). We further probed these results with lamina neuron subtype markers (Chen et al, 2010; Hasegawa et al, 2013; Fernandes et al, 2017) to examine whether loss of *Hbs1* impacted only a subpopulation of L1–L5 neurons which may be obscured by using a pan-neuronal marker. However, these analyses did not reveal any differences in the number of individual L1–L5 subtypes or total number of lamina neurons between *w*^*1118*^ wild-type and *Hbs1* mutants (Figs. 6C and EV5A,B). We also used a pan-glial marker, Repo (Xiong et al, 1994; Halter et al, 1995), and observed no differences between wild-type and *Hbs1* mutants in either the arrangement or numbers of epithelial and marginal glia, which can be distinguished based on their location in the lamina neuropil (Fig. 6A–D). These data led us to consider that the ERG defects in *Hbs1* mutants are unlikely to be caused by disruptions to lamina neuron or glial specification or survival, but instead may arise due to defects in synaptic transmission between the photoreceptors and lamina neurons.

To test for synaptic disruptions in the lamina neuropil, we stained eye-optic lobe preparations with the neuronal membrane marker, HRP (Jan and Jan, 1982). Strikingly, HRP staining in *Hbs1* mutants revealed an increased incidence of vacuoles within the neuropil, visible as dark ‘holes’ in the neuropil (Fig. 6A,E). Vacuolization can arise from defects in synapse formation/maintenance, photoreceptor axon transport, or glial support (Coombe and Heisenberg, 1986; Jackson et al, 2002; Iijima-Ando et al, 2012; Lee and Sun, 2015). However, our ERG analyses do not suggest defects in photoreceptors based on unchanged receptor potential in young animals (Fig. 3), and depleting *Hbs1* in glia did not result in ERG defects (Fig. EV3). Based on these eliminatory analyses and considering all our combined data, we conclude that Hbs1-Pelo likely regulates ATF4 levels in lamina neurons and that such regulation is required for proper synaptic transmission between the lamina and photoreceptor layer.

## Discussion

Inherited retinal diseases are a varied group of genetic disorders that cause progressive vision loss due to gradual photoreceptor death (Duncan et al, 2024). While most of the over 260 genes identified for these diseases are specifically expressed in retinal cells, more broadly expressed ribosome-associated proteins have also been associated with retinal degeneration. Specifically, mutations in RPL10, GTPBP1, GTPBP2, and HBS1L have been shown to result in progressive vision loss, in addition to general neurodegeneration (Zanni et al, 2015; Ishimura et al, 2014; O’Connell et al, 2019; Terrey et al, 2020), with much ongoing research aimed at understanding the molecular underpinnings of these diseases. Our work here uses a *Drosophila* model to significantly advance our etiological understanding of vision defects associated with HBS1L deficiency and provides a preliminary molecular mechanism for progressive vision loss in patients.

### Regulation of translation reinitiation by termination factors

Our investigation of vision defects in *Hbs1* mutants was rooted in our efforts towards understanding regulation of the ISR transcription factor, ATF4. ATF4 is tightly regulated at multiple stages, including transcription, transcript stability, translation, and protein stability. Among these, regulation of mRNA translation within the ATF4 5’ leader is the best characterized and arguably, the most impactful on ATF4 protein levels. The human ATF4 5’ leader has two uORFs, with the final uORF2 overlapping with the ATF4 ORF. A substantial body of work has investigated the signaling events that favor delayed reinitiation at the ATF4 ORF in lieu of reinitiation at uORF2, predominantly the regulation of initiator methionine availability by the ISR pathway. Regardless, it remains that there is at least one, if not two, translation termination events (at the start-stop and at uORF1) within the 5’ leader that precedes reinitiation at the ATF4 start codon. Our data implies a role for efficient ribosome splitting by HBS1L-Pelo at these upstream stop codons (Fig. 2D), which is a necessary precursor to reinitiation at the ATF4 start codon.

High-resolution ribosome profiling data from the Teleman laboratory has convincingly demonstrated that DENR-MCTS1 or eIF2D is required to evacuate spent tRNAs from codons penultimate to the stop codon and thus promote faithful termination (Bohlen et al, 2020). Their meta-analyses further revealed that these factors were specifically required at certain codon identities, including GCG^Ala^ which is the penultimate codon in the uORF1 of the ATF4 mRNA across many species. Such bias towards codon identity for the action of DENR-MCTS1/eIF2D likely stems from their preferential binding to specific tRNAs. Based on the molecular function of HBS1L-Pelo, it is unlikely that penultimate codon identity is relevant to their action on the ATF4 5’ leader. Notably, Pelo was implicated in translation reinitiation of another stress-responsive uORF-containing transcript, C/EBPα (Fernandez et al, 2024). It remains to be investigated whether the presence of uORFs is sufficient for an mRNA to be targeted by HBS1L-Pelo, or whether there are other sequence features in an mRNA that render such selectivity. Further, since reinitiation in uORF-containing mRNAs is enhanced when ISR signaling is activated, it raises the question of whether HBS1L-Pelo activity is more relevant under stress conditions.

Previous mechanistic studies have demonstrated that HBS1L-Pelo promote ribosome recycling on mRNAs that are targeted for no-go decay and no-stop decay, which are mRNA surveillance mechanisms that identify and degrade faulty mRNA transcripts (Shoemaker et al, 2010; Saito et al, 2013). Intriguingly, the *ATF4* mRNA is a known target for another mRNA surveillance mechanism, nonsense-mediated decay (NMD) target (Wang et al, 2011; Wengrod et al, 2013) raising the question of whether the effects of HBS1L-Pelo on ATF4 are linked to its role in NMD. However, our data shows that depleting HBS1L or Pelo does not impact *ATF4* mRNA levels (Fig. EV2A), which would preclude the involvement of mRNA degradation mechanisms such as NMD.

### Selective mRNA translation in the visual system

An increasing body of literature provides evidence that translational regulation drives visual system development to a similar extent as classic transcriptional programs (Jung and Holt, 2011; Zhang et al, 2016; Chen et al, 2021; Ichinose et al, 2024) and others). Under this paradigm, the presence of an mRNA does not necessarily imply that the corresponding protein is translated. Instead, cellular mRNAs are selectively translated due to their sequence features. We propose that in some cases, this selectivity falls to specialized translation factors such as EIF3H, DENR, MCTS1, EIF2D as exemplified by their role in regulating translation reinitiation in the ATF4 5’ leader (Roy et al, 2010; Bohlen et al, 2020; Vasudevan et al, 2020). Our work herein extends this list to HBS1L and Pelo.

Our data showed that *ATF4* mutants exhibit receptor potential defects (Fig. 3G), which we do not observe with *Hbs1* or *pelo* loss of function, implying that other factors are likely required for the proper expression of ATF4 in photoreceptors. Likewise, specialized factors such as HBS1L-Pelo likely have other relevant mRNA targets, both in the visual system and elsewhere. For instance, *Drosophila Hbs1* and *pelo* mutants display defective spermatogenesis, though such a phenotype has not been reported in *ATF4* mutants and it remains unknown which mRNAs downstream of Hbs1-Pelo effect this phenotype. The best way to identify the targets for these specialized factors is through cell-type-specific ribosome profiling, a technically challenging method that has recently yielded promising results (Ichinose et al, 2024). High-resolution translational profiling combined with comparative analyses using model organisms is expected to reveal more such paradigms in the future.

### ISR signaling in visual system development and function

Recent research from multiple groups has implicated the ISR pathway in the visual system, particularly in the context of retinal disorders like autosomal dominant retinitis pigmentosa (adRP) (Bhootada et al, 2016; Athanasiou et al, 2017; Comitato et al, 2019; Vasudevan et al, 2020, 2022; Zhao et al, 2023). Nearly 25% of adRP cases are caused by misfolding-prone mutations in Rhodopsin (Lewin et al, 2014). The prevailing model suggests that misfolded rhodopsin activates the ER stress-sensitive ISR kinase, PERK, which initiates a protective transcriptional program. Predictably, loss of *PERK* exacerbates retinal degeneration in a *Drosophila* adRP model (Vasudevan et al, 2020). The protective function of PERK in photoreceptors is attributed this cell type’s high secretory load of opsins, which makes it particularly reliant on maintaining ER homeostasis (Zhang et al, 2014), amongst other causes. Such a protective role has been extended to other factors downstream of PERK, including ATF4 and its regulator, eIF2D, and also other ER stress response pathways (Vasudevan et al, 2022, 2020; Yan et al, 2019; Coelho et al, 2013; Ryoo et al, 2007). Based on this, we predict that loss of *Hbs1* and *pelo* will likely also result in exacerbated retinal degeneration in the *Drosophila* adRP model.

Our findings here using ERG analyses in *ATF4* mutants (Fig. 3) extends the role of ISR signaling beyond the context of adRP, to visual system development and function. We previously demonstrated that partial loss-of-function mutants of *ATF4* exhibit age-dependent retinal degeneration (Vasudevan et al, 2022). Others have confirmed expression of an ATF4 5’ leader reporter in multiple cell types within the *Drosophila* visual system, including photoreceptors (Kang et al, 2015). In photoreceptors, the role for PERK-ATF4 and other pathways involved in maintaining ER homeostasis appears intuitive due to the highly secretory nature of this cell type. Consistently, our data also shows that loss of ATF4 function results in a decreased receptor potential in young flies (Fig. 3G). However, the specific ATF4 transcriptional targets that contribute to these phenotypes remain unknown. Our data herein also demonstrate a crucial and previously undescribed role for ATF4 in lamina neurons, as evidenced by (1) diminished on- and off-transients in *ATF4* loss of function (Figs. 3E–H and 5A–D), and (2) the rescue of on- and off-transients in *Hbs1* mutants by restoring ATF4 expression (Fig. 5E–H). Whether and how ISR signaling is activated in these second-order neurons during development is an open question, and the molecular role of ATF4 in synapse formation between the lamina and photoreceptors is less intuitive. Notably, lamina neurons are not considered highly secretory or have extensive ER structures like photoreceptors, making speculation of potential ATF4 targets in this cell type difficult. These avenues bear clear pharmacological interest and warrant further investigation.

### Implications of lamina Hbs1 function in human HBS1L deficiency-related vision loss

Our data clearly demonstrate a role for Hbs1 in the lamina, as seen by ERG (Fig. 4) and confocal analyses (Fig. 6). We further narrowed down Hbs1 function to lamina neurons, since depleting Hbs1 in glia using *Rh1-GAL4* or *Repo-GAL4* did not show any ERG defects (Fig. EV4D–I). Interestingly, we do see a small, albeit statistically insignificant decrease in receptor potential in both these datasets, indicative of the onset of age-related defects in photoreceptors and glia, warranting further investigation. Further, it is possible that there are other cell types that require Hbs1-Pelo for their function that our study did not capture. Despite these other potential roles, the involvement of Hbs1 in lamina neurons is made evident by the observed vacuolization defects (Fig. 6).

Both *Drosophila* lamina neurons and human bipolar cells act as interneurons that are responsible for relaying and processing visual information from photoreceptor cells (Sanes and Zipursky, 2010; Malin and Desplan, 2021). In the fly, the R1–R6 photoreceptors terminate in the lamina and synapse with L1–L5 lamina neurons, which then transmit this information to the medulla. Similarly, in the human eye, photoreceptors (rods and cones) synapse with bipolar cells, which then relay the signal to the retinal ganglion cells. Our extensive genetic analyses implicate *Drosophila* Hbs1 and its binding partner, Pelo, in proper function of the lamina neurons (Figs. 3 and 4), specifically in synapse formation between the lamina neurons and photoreceptors (Fig. 5). Extrapolating these analyses to the human visual system, we propose that HBS1L is likely required for proper development of bipolar neurons. There is no direct evidence in other models that loss of Pelo results in vision defects. However, previous work has shown that human HBS1L deficiency patient fibroblasts show lower levels of Pelo protein (O’Connell et al, 2019), suggesting that at least some cell types in HBS1L deficiency patients also have reduced Pelo. This further supports a model wherein HBS1L acts together with Pelo in visual system development and function.

As with the *Drosophila* ERG, the human ERG response can be quantified by the amplitude and timing of specific changes in electrical potential, called the a-wave and b-wave (Gauvin et al, 2018). The a-wave represents the electrical activity of the photoreceptors (rods and cones), indicating their health and function. The subsequent b-wave reflects the activity of the bipolar cells and Müller glia, which are responsible for transmitting the signal from the photoreceptors to the inner retina. Consistent with our conclusion that HBS1L is required primarily in bipolar cells, ERG readings from human HBS1L deficiency patients have been reported to show dampened b-wave amplitudes (Luo et al, 2024). However, human ERG readings from HBS1L deficiency patients also show reduced a-wave amplitudes, indicative of defective photoreceptor (particularly cone cell) function (Luo et al, 2024), which is reminiscent of reduced receptor potential in *Hbs1* mutants with age (Fig. 4I). Thus, we consider that the cone cell impairment in HBS1L deficiency patients is an age-dependent secondary consequence of improper synapse development between photoreceptors and bipolar neurons. Indeed, such a mechanism has been demonstrated in some mouse models of retinal degeneration disorders (Ou et al, 2015; kleine Holthaus et al, 2018) and observed with lamina neuron defects in *Drosophila* (Soukup et al, 2013; Lee and Sun, 2015). This hypothesis is also somewhat supported by retina images from mouse *HBS1L* deletion mutants, which showed fewer photoreceptors at 14 days postnatal but not 7 days postnatal (Luo et al, 2024). Further, whole retina images from *HBS1L* mutant mice show reduced thickness in the outer plexiform layer (Luo et al, 2024), which houses the synapses between photoreceptors and bipolar cells and is the equivalent of the lamina neuropil (Sanes and Zipursky, 2010; Malin and Desplan, 2021). Finally, transcriptomic analyses from the mouse retina show that HBS1L is expressed in bipolar cells and also in photoreceptor cells (Luo et al, 2024). Taking the data across these multiple models together, we speculate that HBS1L-Pelo is required in bipolar cells for proper synapse formation with photoreceptors. Thus, our work herein proffers the first known mechanistic framework for understanding the basis of vision defects in HBS1L deficiency patients.

## Methods

**Table:** Reagents and tools table

Reagent/Resource	Reference or Source	Identifier or Catalog Number
**Experimental models**		
HEK293T (*H. sapiens*)	ATCC	CRL-3216
*Dcg-GAL4 (D. melanogaster)*	Bloomington Drosophila Stock Center (BDSC)	BDSC_7011
*4E-BP* ^*intron*^ *-DsRed (D. melanogaster)*	(Kang et al, 2016)	N/A
*UAS-GFP (D. melanogaster)*	BDSC	BDSC_4776
*UAS-LacZ*^*RNAi*^ *(D. melanogaster)*	(Kang et al, 2016)	N/A
*UAS-Hbs1*^*RNAi*^ *(1) (D. melanogaster)*	Vienna Drosophila Resource Center (VDRC)	v104327
*UAS-Hbs1*^*RNAi*^ *(2) (D. melanogaster)*	VDRC	v33419
*UAS-Elf*^*RNAi*^ *(D. melanogaster)*	VDRC	v106240
*UAS-pixie*^*RNAi*^ *(1) (D. melanogaster)*	VDRC	v109630
*UAS-pixie* ^*RNAi*^ *(2) (D. melanogaster)*	VDRC	v44325
*UAS-Gtpbp1* ^*RNAi*^ *(1) (D. melanogaster)*	VDRC	v27490
*UAS-Gtpbp1*^*RNAi*^ *(2) (D. melanogaster)*	VDRC	v109410
*UAS-Gtpbp2* ^*RNAi*^ *(D. melanogaster)*	VDRC	v107015
*UAS-eRF1*^*RNAi*^ *(D. melanogaster)*	VDRC	v45027
*w*^*1118*^ *(D. melanogaster)*	BDSC	BDSC_3605
*Hbs1*^*1*^ *(D. melanogaster)*	(Yang et al, 2015)	N/A
*Hbs1*^*48*^ *(D. melanogaster)*	(Yang et al, 2015)	N/A
*pelo* ^*1*^ *(D. melanogaster)*	BDSC	BDSC_11757
*pelo*^*PB60*^ *(D. melanogaster)*	BDSC	BDSC_68149
*crc*^*GFSTF*^ *(D. melanogaster)*	BDSC	BDSC_59608
*Gcm-GAL4 (D. melanogaster)*	BDSC	BDSC_45741
*Rh1-GAL4 (D. melanogaster)*	BDSC	BDSC_8391
*Repo-GAL4 (D. melanogaster)*	BDSC	BDSC_7415
*UAS-pelo*^*RNAi*^ *(D. melanogaster)*	VDRC	v108606
*UAS-ATF4*^*RNAi*^ *(D. melanogaster)*	VDRC	v109014
*UAS-LacZ (D. melanogaster)*	BDSC	BDSC_3956
*UAS-ATF4(leaderless) (D. melanogaster)*	(Vasudevan et al, 2020)	N/A
*UAS-ATF4 5’-DsRed (D. melanogaster)*	(Walsh et al, 2025)	N/A
**Recombinant DNA**		
Wild-type ATF4 5’ leader-GFP	Addgene	21852
Mutant ATF4 5’ leader-GFP	Addgene	21863
Wild-type ATF4 5’leader-luciferase	Dr. Aurelio Teleman	N/A
**Antibodies**		
Rabbti anti-ATF4	Cell Signaling	11815S
Rabbit anti-HBS1L	Proteintech	10359-1-AP
Rabbit anti-Pelota	Proteintech	0582-1-AP
Mouse anti-Tubulin	DSHB	12G10
Chicken anti-GFP	Immunology consultants	50-196-2090
Puromycin	Sigma	MABE343
Renilla luciferase	Abcam	ab185926
Rabbit anti-Phosphorylated eIF2a	Cell Signaling	9721
Rabbit anti-Total eIF2a	Cell Signaling	5324
Donkey anti-rabbit HRP	Thermo Fisher Scientific	SA1200
Goat anti-mouse HRP	Thermo Fisher Scientific	A16066
Goat anti-chicken HRP	Thermo Fisher Scientific	A16054
StarBright Blue 700 Goat Anti-Mouse	BioRad	12004158
Rat anti-Elav	DSHB	7E8A10
Mouse anti-Repo	DSHB	8D12
DyLight 405 conjugated goat anti-HRP	Jackson ImmunoResearch	123-475-021
Guinea pig anti-Slp2	Dr. Claude Desplan	N/A
Mouse anti-Svp	DSHB	2D3
Rat anti-Erm	Dr. Claude Desplan	N/A
Rabbit anti-Bsh	Dr. Claude Desplan	N/A
Alexa Rhodamine Red-X anti-rat	Jackson ImmunoResearch	712-295-153
Alexa Fluor 647 anti-mouse	Jackson ImmunoResearch	715-605-151
Alexa Fluor 488 anti-guinea pig	Jackson ImmunoResearch	706-545-148
Alexa Fluor Rhodamine Red-X anti-mouse	Jackson ImmunoResearch	715-295-151
Alexa Fluor 647 anti-rat	Jackson ImmunoResearch	712-605-153
Alexa Fluor 405 anti-rabbit	Invitrogen	A48258
**Oligonucleotides and other sequence-based reagents**		
Control siRNA (negative control)	Thermo Fisher Scientific	4390843
*HBS1L* siRNA (Thermo Silencer Select)	Thermo Fisher Scientific	s21152
Pelo siRNA (Thermo Silencer Select)	Thermo Fisher Scientific	s28807
ATF4 qPCR primers	IDT	F:GGAGATAGGAAGCCAGACTACA, R: GGCTCATACAGATGCCACTATC
GAPDH qPCR primers	IDT	F:GTCTCCTCTGACTTCAACAGCG, R:ACCACCCTGTTGCTGTAGCCAA
**Chemicals, Enzymes and other reagents**		
16% paraformaldehyde, EM grade	Fisher Scientific	50980487
4′,6-diamidino-2-phenylindole (DAPI, 200 μM final)	Fisher Scientific	574810
70% glycerol	Genesee Scientific	18-205
SlowFade	Life Technologies	S36917-5X2ML
high glucose Dulbecco’s Minimal Essential Medium (DMEM)	Thermo Fisher Scientific	11965092
Fetal Bovine Serum (heat inactivated)	Thermo Fisher Scientific	16140071
Penicillin/Streptomycin	Thermo Fisher Scientific	15140122
Lipofectamine 2000	Invitrogen	11668027
Radioimmunoprecipitation assay (RIPA) buffer	N/A	As described in the Methods section
Laemmli buffer	N/A	As described in the Methods section
SuperSignal West Pico PLUS Chemiluminescent Substrate,	Thermo Fisher Scientific	34577
BSA	Fisher Scientific	BP9703100
Puromycin	Thermo Fisher Scientific	J67236.8EQ
Trizol	Invitrogen	15596018
Thermo Maxima reverse transcriptase	Thermo Fisher Scientific	FEREP0741
SYBR Green	Mid Sci	PR2000-N-25BLK
Triton X-100	Millipore Sigma	T8787
Tween-20	Millipore Sigma	P9416
**Software**		
ImageJ v2.1.0/1.53c	https://imagej.net/ij/index.html	N/A
LabChart 8	AD Instruments	N/A
GraphPad Prism 11.0.1	https://www.graphpad.com/	N/A
**Other**		
A1 inverted line scanning confocal microscope	Nikon	N/A
SP8 upright point scanning confocal	Leica	N/A
Electrophysiology rig covered with a black out Faraday cage	Vilinsky and Johnson, 2012; Wu et al, 2022; Meece et al, 2025	N/A
Standard electrode holder	Warner Instruments E series	
Glass capillaries	World Precision Instruments	model 1B150F-4
Vertical pipette puller	David Kopf instruments	
Micromanipulators	World Precision Instruments	
Amplifier	A/M Systems	Headstage Model 3000 (Regular)
A/D converter	AD Instruments	PowerLab 4/30
Blue LED	Phillips Luxeon Rebel LED	Wavelength: 470 ± 20 nm

### *Drosophila* husbandry and stocks

All stocks and crosses were reared at 25 °C with a 12-h light/dark cycle. Progeny of interest were collected within 2 days of eclosion and aged in vials with no more than 10 animals each aged at 25 °C. Aging animals were transferred to new vials every 3 days to ensure fresh food availability. Animals were raised on ‘R’ food formulation from Lab Express (https://www.lab-express.com/DIS58.pdf). All fly stocks used in the study, and the specific figures wherein they are employed are listed in Table 2.

**Table 2:** *Drosophila* stocks used in this study.

Transgene	Source	Figure
*Dcg-GAL4*	BDSC_7011	1A,B; EV2D,E
*4E-BP*^*intron*^*-DsRed*	(Kang et al, 2016)	1A–F; EV1
*UAS-GFP*	BDSC_4776	1A
*UAS-LacZ*^*RNAi*^	(Kang et al, 2016)	1A,B; 4A–E; 5A–D; EV2D,E; EV4D–I
*UAS-Hbs1*^*RNAi*^ *(1)*	v104327	1A,B
*UAS-Hbs1*^*RNAi*^ *(2)*	v33419	1A,B; 4A–E; EV2D,E; EV4D–I
*UAS-Elf*^*RNAi*^	v106240	
*UAS-pixie*^*RNAi*^ *(1)*	v109630	
*UAS-pixie*^*RNAi*^*(2)*	v44325	
*UAS-Gtpbp1*^*RNAi*^*(1)*	v27490	
*UAS-Gtpbp1*^*RNAi*^ *(2)*	v109410	
*UAS-Gtpbp2* ^*RNAi*^	v107015	
*UAS-eRF1*^*RNAi*^	v45027	
*w*^*1118*^	BDSC_3605	1C–F; 3A,E–G; 5E–H; 6A–E; EV1A,B; EV4A–C; EV5A,B
*Hbs1*^*1*^	(Yang et al, 2015)	1C,D; 3B,C,E–G; 5E–H; 6A–E; EV1A; EV4A–C; EV5A,B
*Hbs1*^*48*^	(Yang et al, 2015)	1C,D; 3B,E–G; 5E–H; 6A–E; EV1A; EV4A–C; EV5A,B
*pelo*^*1*^	BDSC_11757	1E,F; 3C,E–G; EV1B; EV4A–C
*pelo*^*PB60*^	BDSC_68149	1E,F; EV1B
*crc*^*GFSTF*^	BDSC_59608	3D–G
*Gcm-GAL4*	BDSC_45741	4A–E; 5A–H
*Rh1-GAL4*	BDSC_8391	EV4D,E
*Repo-GAL4*	BDSC_7415	EV4G–I
*UAS-pelo*^*RNAi*^	v108606	5A–D; EV2D,E
*UAS-ATF4*^*RNAi*^	v109014	5A–D
*UAS-LacZ*	BDSC_3956	5E–H
*UAS-ATF4(leaderless)*	(Vasudevan et al, 2020)	5E–H
*UAS-ATF4 5’-DsRed*	(Walsh et al, 2025)	EV2D,E

Sex as a biological variable: Initial observations were recorded in both males and females to ensure that the phenotypes were not sexually dimorphic. All the final quantified analyses performed in this study utilize male animals in both larval and adult experiments for experimental consistency.

### Immunostaining and confocal microscopy

Fat bodies from wandering third instar male larva were dissected in 1X phosphate buffer saline (PBS) and placed in an Eppendorf tube. Tissues were fixed in 4% paraformaldehyde (PFA), 1X PBS for 20 min on a nutator, and washed twice for 10 min each in 0.1% PBS-Tween (PBST). Samples were incubated for 10 min in the dark with 4′,6-diamidino-2-phenylindole (DAPI, 200 μM final) to counterstain for nuclei. Fat bodies were finally mounted on glass slides in a solution of 70% glycerol prior to placing coverslips. Slides were sealed with a thin coating of nail polish to prevent evaporation and imaged on a Nikon A1 inverted line scanning confocal microscope.

Adult whole brains (optic lobes and central brain) were dissected in 1X PBS and placed in a glass dish, where they were fixed in 4% PFA for 30 min. Samples were washed with 0.5% PBTx (1X PBS with 0.5% TritonX) for at least 1 h, and then incubated with primary antibodies diluted in block solution (5% normal horse serum in PBTx) for 48 h at 4 °C. After washing with 0.5% PBTx, samples were further incubated for 48 h at 4 °C with secondary antibodies diluted in block, washed again, and finally mounted in SlowFade (Life Technologies). Slides were imaged on a Leica SP8 upright point scanning confocal, with 40× objective and 2× zoom, and stacks were acquired with a step size of 1 μm.

#### Image quantification

##### Fat body

Images were analyzed in ImageJ v2.1.0/1.53c. Using the DAPI channel and the ‘Threshold’ function, individual nuclei were identified as regions of interest (ROI). DsRed intensity was then collected for each ROI and graphed.

##### Lamina

Images were analyzed in ImageJ v2.1.0/1.53c. The Cell counter plug-in was used for lamina neuron subtype counts. Svp, Slp2 double positive cells were counted as L1 neurons. Slp2 single positive cells were counted as L2 neurons. Erm-positive cells were counted as L3 neurons. Bsh positive cells were counted as L4 neurons, and Slp2, Bsh double positive cells were counted as L5 neurons. The number of cells were counted in each z-stack slice and averaged across equal number of slices for every sample to create a single data point for each sample. The sum of the lamina neuron subtypes were calculated for the total lamina neuron counts. Epithelial glial counts were done with ImageJ cell counter plug-in and were identified as Repo-positive cells distal to the lamina neuropil, while marginal glia are Repo-positive cells proximal to the lamina neuropil. Percent vacuolization was quantified by the sum of the areas of each vacuole (regions lacking HRP staining) in one neuropil divided by the total area of the neuropil. Each data point represents the average percent vacuolization across z stacks from one animal.

### Cell culture and western blot analyses

HEK293T cells were cultured in high-glucose Dulbecco’s minimal essential medium (DMEM) supplemented with 10% Fetal Bovine Serum and 1% Penicillin/Streptomycin. siRNA and plasmid (catalog details below) co-transfections were performed using Lipofectamine 2000 (Life Technologies) according to manufacturer’s protocol in 6-well dishes. 48 h after transfection, cells were washed twice in 1x PBS and lysed in 100 μl of Radioimmunoprecipitaton assay (RIPA) buffer (50 mM Tris-HCl pH 7.4, 150 mM NaCl, 1% Triton X-100, 0.5% Sodium deoxylcholate, 0.1% SDS, 1 mM EDTA, protease inhibitor cocktail), and the lysate was cleared by centrifuging for 10 min at 14,000 rcf at 4 °C. 20 μl of the lysate was mixed with the appropriate amount of 4x Laemmli buffer (277.8 mM Tris-HCl pH 6.8, 40% v/v glycerol, 4% SDS, 0.02% bromophenol blue, 4% β-mercaptoethanol) and analyzed by western blotting on nitrocellulose membrane. Membranes were blocked for 1 h at room temperature in 5% non-fat dry milk diluted in PBST. They were then incubated in primary antibodies (listed below) diluted in 5% BSA in PBST overnight at 4 °C. Following three 10-min washes in PBST, membranes were incubated in secondary antibodies for 2 h at room temperature and washed thrice for 10 min in PBST before HRP detection.

#### Puromycin assays

Cells transfected as above were treated with 10 μg/ml for 15 min prior to lysis with RIPA buffer (as above). Lysates were then analyzed by western blotting as described above.

#### qPCR

Cells transfected as above were washed twice with 1x PBS and total RNA was collected by adding Trizol (Life Technologies) directly to the cell culture dish following manufacturer’s protocol for RNA preparation. 1000 ng of RNA was used in a 20 μl cDNA reaction using the Thermo Maxima reverse transcriptase (Life Technologies) following manufacturer’s protocol. Quantitative RT-PCR analysis was performed using SYBR Green (Mid Sci) and relevant primers listed below.

### ERG recordings and analyses

All ERG recordings were performed in an electrophysiology rig covered with a black out Faraday cage following a protocol derived from prior studies (Vilinsky and Johnson, 2012; Wu et al, 2022; Meece et al, 2025). *Drosophila* were anesthetized by placing them on ice for 10 min and immobilized using dental wax on glass coverslips for electrophysiological recordings as described before (Meece et al, 2025). Reference and recording electrodes were prepared as in Vilinsky and Johnson (2012) and clamped into standard electrode holder (Warner Instruments E series, straight configuration). The reference electrode was generated using glass capillaries (World Precision Instruments, model 1B150F-4) in vertical pipette puller (David Kopf Instruments) to approximate tip size of 0.5μm and filled with 0.9% w/v NaCl solution. The recording electrode is generated by inserting a cotton sewing thread into the barrel of an (un-pulled) glass capillary then filled with 0.9% w/v NaCl. Using two micromanipulators (World Precision Instruments), the reference electrode was inserted in the thorax just below the wing, and the recording electrode was gently placed on the external eye. The recording electrode was connected to the input headstage of an A/M Systems headstage Model 3000 (Regular) amplifier, and the reference electrode was connected to a common aluminum ground bar located inside the Faraday cage. Data from the amplifier were routed to an A/D converter (AD Instruments PowerLab 4/30) and subsequently acquired, analyzed, and displayed using the program LabChart 8 (AD Instruments). Animals were stimulated with a 1 s exposure to blue LED (wavelength: 470 ± 20 nm, Philips Luxeon Rebel LED). Each animal was measured three times, with a one-minute rest between recordings. All ERG analyses were performed from animals collected across at least two independent crosses reared in identical conditions, using similar ERG setups.

### Statistical analysis

The figure legends describe the sample sizes for the corresponding data. Multiple comparisons with a control were made using a Kruskal–Wallis test with Dunn’s correction for multiple comparisons. Paired comparisons were made using the Mann–Whitney test for nonparametric data in all instances when we could not assume Gaussian distribution. For data where control samples were normalized to 1 (such as western blots or qPCRs), a Wilcoxon signed rank test was conducted when comparing a given sample to the control. These datasets have matched data, where each data point has a corresponding measurement across all conditions, thus when making multiple comparisons, we utilized a Friedman test for nonparametric data with Dunn’s correction for multiple comparisons. When comparing two conditions in these datasets, we used a Wilcoxon matched-pairs signed rank.

## Supplementary information

Peer Review File

Source data Fig. 1

Source data Fig. 2

Source data Fig. 3

Source data Fig. 4

Source data Fig. 5

Source data Fig. 6

Expanded View Figures

## Data Availability

This study includes no data deposited in external repositories. The source data of this paper are collected in the following database record: biostudies:S-SCDT-10_1038-S44319-026-00882-6.
