# Supplementary material for: Visual system function requires translational regulation of ATF4 by Hbs1-Pelo
Source: EMBO Rep. 2026 Jul 17;27(17):5121–42. doi: 10.1038/s44319-026-00882-6 (PMC13554125; doi:10.1038/s44319-026-00882-6)
Supplement: Supplementary file 1 [file 44319_2026_882_MOESM1_ESM.pdf]

EMBO reports

Visual system function requires translational regulation of ATF4 by Hbs1-Pelo

Katherine Tempro, Inês Lago-Baldaia, Narayanan Nampoothiri V.P., Christopher Garbark, Abby Carney, Vilaiwan Fernandes, and Deepika Vasudevan

Corresponding author(s): Deepika Vasudevan (deepikav@pitt.edu)

Review Timeline:

Submission Date:	16th Oct 25
Editorial Decision:	5th Dec 25
Revision Received:	6th Apr 26
Editorial Decision:	29th Apr 26
Revision Received:	7th Jul 26
Accepted:	9th Jul 26

Editor: Achim Breiling

Transaction Report:

(Note: With the exception of the correction of typographical or spelling errors that could be a source of ambiguity, letters and reports are not edited. Depending on transfer agreements, referee reports obtained elsewhere may or may not be included in this compilation. Referee reports are anonymous unless the Referee chooses to sign their reports.)

1st Editorial Decision **5th Dec 2025**

Dear Dr. Vasudevan,

Thank you for the submission of your manuscript to EMBO reports. I have now received reports from the three referees that were asked to evaluate your study, which can be found at the end of this email. As you will see, the referees think that these findings are of interest. However, they have several comments, concerns, and suggestions, indicating that a major revision of the manuscript is necessary to allow publication of the study in EMBO reports.

As the reports are below, and all the referee concerns need to be addressed, I will not detail them here. However, I consider experiments regarding point 7 of referee #7 out of scope for this study. Thus, this point does not need to be addressed experimentally during revision.

Given the constructive referee comments, I would thus like to invite you to revise your manuscript with the understanding that the concerns of the referees must be addressed in the revised manuscript and/or in a detailed point-by-point response. Acceptance of your manuscript will depend on a positive outcome of a second round of review. It is EMBO reports policy to allow a single round of revision only and acceptance of the manuscript will therefore depend on the completeness of your responses included in the next, final version of the manuscript.

Revised manuscripts should be submitted within three months of a request for revision. Please contact me to discuss the revision (also by video chat) if you have questions or comments regarding the revision, or should you need additional time.

When submitting your revised manuscript, please also carefully review the instructions that follow below.

PLEASE NOTE THAT upon resubmission revised manuscripts are subjected to an initial quality control prior to exposition to re-review. Upon failure in the initial quality control, the manuscripts are sent back to the authors, which may lead to delays. Frequent reasons for such a failure are the lack of the data availability section (please see below) and the presence of statistics based on n=2 (the authors are then asked to present scatter plots or provide more data points).

When submitting your revised manuscript, we will require:

1) a .docx formatted version of the final manuscript text (including legends for main figures, EV figures and tables), but without the figures included. Figure legends should be compiled at the end of the manuscript text.

2) individual production quality figure files as .eps, .tif, .jpg (one file per figure), of main figures and EV figures. Please upload these as separate, individual files upon re-submission.

The Expanded View format, which will be displayed in the main HTML of the paper in a collapsible format, has replaced the Supplementary information. You can submit up to 6 images as Expanded View. Please follow the nomenclature Figure EV1, Figure EV2 etc. The figure legend for these should be included in the main manuscript document file in a section called Expanded View Figure Legends after the main Figure Legends section. Additional Supplementary material should be supplied as a single pdf file labeled Appendix. The Appendix should have page numbers and needs to include a table of content on the first page (with page numbers) and legends for all content. Please follow the nomenclature Appendix Figure Sx, Appendix Table Sx etc. throughout the text, and also label the figures and tables according to this nomenclature.

For more details, please refer to our guide to authors:
https://link.springer.com/journal/44319/submission-guidelines#cms-Revised-submissions

Please consult our guide for figure preparation:
https://media.springernature.com/original/springer-cms/rest/v1/content/27825798/data/v1

3) a .docx formatted letter INCLUDING the reviewers' reports and your detailed point-by-point responses to their comments. As part of the EMBO Press transparent editorial process, the point-by-point response is part of the Review Process File (RPF), which will be published alongside your paper.

4) a complete author checklist, which you can download from our author guidelines (https://link.springer.com/journal/44319/submission-guidelines#cms-Revised-submissions). Please insert page numbers in the checklist to indicate where the requested information can be found in the manuscript. The completed author checklist will also be part of the RPF.

Please also follow our guidelines for the use of living organisms, and the respective reporting guidelines:
https://link.springer.com/partners/embo-press/editorial-policies#Animal%20and%20human%20studies

5) that primary datasets produced in this study (e.g. RNA-seq, ChIP-seq, structural and array data) are deposited in an

appropriate public database. If no primary datasets have been deposited, please also state this in a dedicated section (e.g. 'No primary datasets have been generated and deposited'), see below.

See also: https://link.springer.com/partners/embo-press/editorial-policies#Data%20deposition

Please remember to provide a reviewer password if the datasets are not yet public.

The accession numbers and database should be listed in a formal "Data Availability" section that follows the model below. This is now mandatory (like the COI statement). Please note that the Data Availability Section is restricted to new primary data that are part of this study. This section is mandatory. As indicated above, if no primary datasets have been deposited, please state this in this section

Data availability

The datasets produced in this study are available in the following databases:

- RNA-Seq data: Gene Expression Omnibus GSE46843 (https://www.ncbi.nlm.nih.gov/geo/query/acc.cgi?acc=GSE46843)
- [data type]: [name of the resource] [accession number/identifier/doi] ([URL or identifiers.org/DATABASE:ACCESSION])

*** Note - All links should resolve to a page where the data can be accessed. ***

See also: https://link.springer.com/partners/embo-press/editorial-policies#Data%20availability%20statement

Moreover, I have these editorial requests:

6) We now request the publication of original source data with the aim of making primary data more accessible and transparent to the reader. You will receive a separate email with instructions for providing source data with your revised manuscript, including information how to upload and organize the files.

7) Our journal encourages inclusion of *data citations in the reference list* to directly cite datasets that were re-used and obtained from public databases. Data citations in the article text are distinct from normal bibliographical citations and should directly link to the database records from which the data can be accessed. In the main text, data citations are formatted as follows: "Data ref: Smith et al, 2001" or "Data ref: NCBI Sequence Read Archive PRJNA342805, 2017". In the Reference list, data citations must be labeled with "[DATASET]". A data reference must provide the database name, accession number/identifiers and a resolvable link to the landing page from which the data can be accessed at the end of the reference. Further instructions are available at: https://link.springer.com/journal/44319/submission-guidelines#cms-Reference-guidelines

8) Regarding data quantification and statistics, please make sure that the number "n" for how many independent experiments were performed, their nature (biological versus technical replicates), the bars and error bars (e.g. SEM, SD) and the test used to calculate p-values is indicated in the respective figure legends (also for EV and Appendix figures). Please also check that all the p-values are explained in the legend, and that these fit to those shown in the figure. Please provide statistical testing where applicable. Please avoid the phrase 'independent experiment' but clearly state if these were biological or technical replicates. Please also indicate (e.g. with n.s.) if testing was performed, but the differences are not significant. In case n=2, please show the data as separate datapoints without error bars and statistics. See also: https://link.springer.com/journal/44319/submission-guidelines#cms-Figure-and-data-presentation

If n<5, please show single datapoints for diagrams.

9) Please add scale bars of similar style and thickness to all microscopic images, using clearly visible black or white bars (depending on the background). Please place these in the lower right corner of the images themselves. Please do not write on or near the bars in the image but define the size in the respective figure legend.

10) Please also note our reference format:
https://link.springer.com/journal/44319/submission-guidelines#cms-Reference-guidelines

11) We updated our journal's competing interests policy in January 2022 and request authors to consider both actual and perceived competing interests. Please review the policy https://www.embopress.org/competing-interests and update your competing interests if necessary. Please name this section 'Disclosure and Competing Interests Statement' and put it after the Acknowledgements section.
See: https://link.springer.com/partners/embo-press/editorial-policies#Competing%20interest%20disclosures

12) We now use CRediT to specify the contributions of each author in the journal submission system. CRediT replaces the author contribution section. Please use the free text box to provide more detailed descriptions and do NOT provide your final manuscript text file with an author contributions section. See also our guide to authors (section 'Author contributions'):

https://link.springer.com/journal/44319/submission-guidelines#cms-Revised-submissions

13) All Materials and Methods need to be described in the main text using our 'Structured Methods' format, which is required for all research articles. According to this format, the Methods section should include a Reagents and Tools Table (listing key reagents, experimental models, software, and relevant equipment and including their sources and relevant identifiers), uploaded as separate file, and a Methods section in which we encourage the authors to describe their methods using a step-by-step protocol format with bullet points, to facilitate the adoption of the methodologies across labs. More information on how to adhere to this format as well as downloadable templates (.doc) for the Reagents and Tools Table can be found in our author guidelines (section 'Structured Methods'):

https://link.springer.com/journal/44319/submission-guidelines#cms-Manuscript-organisation-and-formatting

14) Please order the manuscript sections like this, using only these names:
Title page - Abstract (max. 175 words) - Keywords - Introduction - Results - Discussion - Methods - Data availability section - Acknowledgements (please include here all the funding information) - Disclosure and Competing Interests Statement - References - Figure legends - Expanded View Figure legends

15) Please make sure that all the funding information is also entered into the online submission system and that it is complete and similar to the one in the acknowledgement section of the manuscript text file.

We would also welcome the submission of cover suggestions, or motifs to be used by our Graphics Illustrator in designing a cover.

As part of the EMBO publication's Transparent Editorial Process, EMBO reports publishes online a Review Process File (RPF) to accompany accepted manuscripts. This File will be published in conjunction with your paper and will include the referee reports, your point-by-point response and all pertinent correspondence relating to the manuscript.

You are able to opt out of this by letting the editorial office know (emboreports@embo.org). If you do opt out, the Review Process File link will point to the following statement: "No Review Process File is available with this article, as the authors have chosen not to make the review process public in this case."

I look forward to seeing a revised form of your manuscript when it is ready.

Yours sincerely,

Achim Breiling
Senior Editor
EMBO Reports

Referee #1:

This is a very nice manuscript which identifies ATF4 as a key mediator of the neurological defects that result from loss of Pelo/Hbs1. It is known that loss of HBS1L in humans causes retinal degeneration, but the underlying mechanism is not understood. This manuscript 1) sets up Drosophila as a model to study loss of Pelo and Hbs1 in vivo, 2) discovers that loss of Pelo/Hbs1 leads to loss of ATF4, and then 3) goes on to show, impressively, that the retinal defects caused by loss of Hbs1 can be rescued by bringing back ATF4 expression.

One strength of this manuscript is that it links everything from loss of Hbs1 to the molecular mechanism to the electrophysiological consequences, ie from the molecular defect to the organismal phenotype. Furthermore, ATF4 is a key, central regulator of a cell's response to stress, which is generally relevant for health and disease, including cancer. Hence understanding how ATF4 translation is regulated is important. This manuscript contributes to our understanding of ATF4 translation by identifying a new translation factor that affects it. Finally, this work has possible relevance for patients by identifying a molecular mechanism that in the future should be analyzed in mammalian systems.

Overall, I think this work will be of interest to a broad audience studying cell stress, mRNA translation, and neurodegeneration.

I have one major suggestion:
Most of the story is assembled in Drosophila, but the mechanistic work linking Pelo/Hbs1 to ATF4 is done in human cells. it would be good to close this mechanistic gap in Drosophila, to have one complete story in the fly. ie it would be good to show that:

1. Also in Drosophila Pelo regulates ATF4 protein levels. As far as I can tell, in Drosophila the data that are shown are a 4EBP reporter that senses Pelo activity, but ATF4 protein levels are not shown? (Or is there no antibody available to detect Drosophila ATF4? Is there a GFP-fusion reporter?)

2. Also in Drosophila Pelo regulates ATF4 translation via its 5'UTR (by cloning a reporter with the dATF4 5'UTR and either putting it in the fly or in Drosophila S2 cells and knocking down Pelo or Hbs1).

Minor issues:
1. The abstract needs some proofreading. e.g.:
" Deletion mutations in the translation termination factor, HBS1L, result in..." should not have commas around HBS1L

" Mechanistically, we foud that..." spelling/typo

In the intro " Loss of ATF4 has been linked to a plethora of developmental abnormalities in the skeletal system, hematopoiesis, adipose and liver tissues"
Hematopoiesis should be "hematopoietic system" because "hematopoiesis" is not a tissue.

2. Fig 1A - In the overlay image is the GFP[nls] shown green or blue? Because it looks green in the image but blue in the labeling above the image. Also, I can clearly see the reduction in the 4E-BP reporter in the single-channel images, but it is difficult to see in the overlay - can the purple be made more clear? Or pick another color?

3. Fig 2C - is the 'control reporter' a reporter with the wildtype ATF4 5'UTR as shown in Fig 2D ? If so, it would be helpful to label the reporters in the schematic in Fig 2C as "control" and "mutant" to make this more clear. (In the Results text it's being referred to as "ATF4 5'-GFP reporter" instead of a control reporter.) This is particularly confusing because its levels do not increase in response to tunicamycin. If this is due to perdurance of the GFP, it would be worth replacing the GFP with either a destabilized GFP or luciferase in order to get a reporter that faithfully reproduces the regulation seen with endogenous ATF4.

4. The drop in global translation rates upon Pelo depletion specifically in the Tunicamycin condition is interesting because it suggests that Pelo activity is more required when eIF2-alpha is phosphorylated. Perhaps it is worth discussing this?

5. The sentence " our ERG analyses revealed that Gcm>LacZ expression resulted in on- and off-transient defects in Hbs11/Hbs148 animals when compared to control w1118 animals (Fig. 5E-G)" is a bit confusing because, if I understand correctly, it's not the LacZ expression that results in the defect, but the Hbs1 mutation.

Referee #2:

The paper by Querry et al. describes how the loss of the ribosome rescue factors Hbs1 or Pelo in Drosophila result in the loss of ATF4. The same appears to be true in human cell lines using knockdown of HBS1L and PELO. The authors examine retinal function in Drosophila models with knockdown or mutations in Hbs1, Pelo, and Atf4/crc, given the optic phenotypes of patients with mutations in these genes. They find a variety of ERG problems that primarily point to neurons in the medulla, although photoreceptor degeneration/dysfunction is also involved in some genotypes and at later ages. Importantly, the ERG phenotype of Hbs1 mutants can largely be rescued by expression of Atf4 without its normal regulatory 5' end.

Overall, the paper is well written, the data are clear and compelling, and the results are well quantified. A few additions would further strengthen the manuscript.

The rescue of Hbs1 mutant ERGs with expression of Atf4 is interesting, but also unexplained. It is possible that unregulated expression of Atf4 is compensating for or otherwise circumventing the loss Hbs1. The extent to which the loss of Atf4 is central to the ERG phenotypes of Pelo and Hbs1 knockdown (3C, 4A, 5A) should be tested by knockdown or conditional knockdown of Atf4 in those backgrounds to see if there is an additional effect.

Minor points:
In results of figure 5, the authors use the term "ATF4 expression." As a general rule, genes are expressed and proteins are at "levels" of "abundance". It seems the authors are referring to ATF4 protein levels, not gene/mRNA expression levels, so "expression" may not be the best descriptor.

In figures 1 and S1, I am unfamiliar with fat body anatomy, can the authors please provide a brief description.

The Discussion section gets a bit speculative on connections between Drosophila retinal phenotypes and the human disease. I suggest tightening up those comparisons and spending additional time on how ATF4, as a transcription factor, may be manifesting these effects. Are there known ATF4 target genes that could contribute, etc. Similarly, is chronically high ATF4 anticipated to be deleterious?

Referee #3:

This manuscript reports a requirement for the ribosome rescue factors Hbs1 and Pelota in development of visual system neurons. It is first reported that loss of either of these rescue factors, which function together as a heterodimer, reduces integrated stress response (ISR) transcription in Drosophila cells. The ISR is driven by the translationally-regulated transcription factor Atf4, and depletion of Hbs1 or Pelo also reduces Atf4 protein levels in human cells. Building on the published observation that human disease mutations and mouse genetic models lacking Hbs1 show visual defects, the effects of these mutants are tested in the Drosophila visual system. Genetic disruption of Hbs1, Pelo, or Atf4 greatly disrupt the transient and steady-state electrical responses to light in Drosophila photoreceptors. These altered responses are partially reproduced by cell type-specific depletion of Hbs1, Pelo, or Atf4 in lamina neurons downstream of photoreceptors. Furthermore, Atf4 overexpression in these lamina neurons can partially suppress Hbs1 depletion. No dramatic changes in cell or tissue organization were seen in Hbs1-depleted lamina, however.

This manuscript describes interesting consequences of Hbs1 depletion in Drosophila. From a molecular perspective, the role for these factors in Atf4 expression is interesting. Likewise, the impact of these pathways on the visual system is notable. However, the connection between these observations is tenuous and neither is analyzed in depth. Furthermore, in both cases, the lack of deeper analysis raises the possibility that indirect and uninformative effects could explain the observed phenomena. My sense is that reasonable revisions could not address these fundamental concerns with the current manuscript. For these reasons, I recommend against publication, with my specific concerns enumerated below.

1. Loss of Hbs1 and Pelo could indirectly activate the ISR. Loss of HBS1L causes global shifts in protein synthesis and altered mTOR signaling (O'Connell et al., 2019), and similar effects could likewise alter ISR activation. Further mechanistic analysis, such as analysis of eIF2α phosphorylation and its regulation, could be valuable for addressing this broad concern.

2. Related to this point, the lack of regulated translation limits the value of 5′ UTR reporter used in Figure 2.

3. It is hard to reconcile the apparent band intensities in Figure 2A with the quantitation in Figure 2C. In Figure 2A, the "GFP" blot has notably faint bands for HBS1L Tu- and both Pelo lanes relative to the others, and quite consistent "Tub" control. The quantitation in Figure 2C shows no difference in HBS1L +/- Tunicamycin, however, and only a modest ~2-fold reduction in the Pelo samples.

4. It is written that, "knockdown of Pelo resulted in a small but statistically significant reduction in the mutant reporter." Why is this not shown on the graph with significance marks?

5. In Figure 3C, a pelo; Hbs1 double heterozygote is analyzed along with a biallelic Hbs1 mutant and an Atf4 heterozygote. Was any analysis performed of single heterozygotes for either pelo or Hbs1?

6. Lamina neuron depletion of Hbs1 (Figure 4) has a weaker and age-dependent effect relative to the global effects of Hbs1 depletion. Were photoreceptor-specific depletions tested?

7. More broadly, what sensitizes laminar neurons in particular to this defect in protein synthesis? Photoreceptor cells have highly specialized physiology and consequently display some particular sensitivities to protein folding and processing. Other highly specialized secretory cells depend particularly on the ISR. Why these laminar neurons?

<u>Response to referee comments (EMBOR-2025-62980V2)</u>

We thank the editor and all the referees for their positive reception of our manuscript and their constructive feedback. We have responded to all referee concerns below. Text changes in the revised manuscript are highlighted in yellow and all figure numbers below reference the revised manuscript.

Referee #1:
This is a very nice manuscript which identifies ATF4 as a key mediator of the neurological defects that result from loss of Pelo/Hbs1. It is known that loss of HBS1L in humans causes retinal degeneration, but the underlying mechanism is not understood. This manuscript 1) sets up Drosophila as a model to study loss of Pelo and Hbs1 in vivo, 2) discovers that loss of Pelo/Hbs1 leads to loss of ATF4, and then 3) goes on to show, impressively, that the retinal defects caused by loss of Hbs1 can be rescued by bringing back ATF4 expression.

One strength of this manuscript is that it links everything from loss of Hbs1 to the molecular mechanism to the electrophysiological consequences, ie from the molecular defect to the organismal phenotype. Furthermore, ATF4 is a key, central regulator of a cell's response to stress, which is generally relevant for health and disease, including cancer. Hence understanding how ATF4 translation is regulated is important. This manuscript contributes to our understanding of ATF4 translation by identifying a new translation factor that affects it. Finally, this work has possible relevance for patients by identifying a molecular mechanism that in the future should be analyzed in mammalian systems.

Overall, I think this work will be of interest to a broad audience studying cell stress, mRNA translation, and neurodegeneration. I have one major suggestion: Most of the story is assembled in Drosophila, but the mechanistic work linking Pelo/Hbs1 to ATF4 is done in human cells. it would be good to close this mechanistic gap in Drosophila, to have one complete story in the fly. ie it would be good to show that:
We thank the reviewer for their positive assessment of our manuscript and their suggestions, which we believe have improved our manuscript.

1. Also in Drosophila Pelo regulates ATF4 protein levels. As far as I can tell, in Drosophila the data that are shown are a 4EBP reporter that senses Pelo activity, but ATF4 protein levels are not shown? (Or is there no antibody available to detect Drosophila ATF4? Is there a GFP-fusion reporter?)2. Also in Drosophila Pelo regulates ATF4 translation via its 5'UTR (by cloning a reporter with the dATF4 5'UTR and either putting it in the fly or in Drosophila S2 cells and knocking down Pelo or Hbs1).
>We agree that demonstrating the mechanism in *Drosophila* improves the comprehensiveness of the manuscript. Unfortunately, there are no reliable antibodies to detect *Drosophila* ATF4. We tried a GFP-fusion ATF4 (PMID 34919148) but found the expression to be very variable across even control animals making it difficult to assess any change with *Hbs1* or *pelo* depletion. Instead, we opted to use a *Drosophila* ATF4 5'-DsRed reporter (PMID 41436469) in the fat body and examined changes with *Hbs1* and *Pelo* RNAi. Consistent with our data in human cells, we found that depleting either factor resulted in decreased DsRed expression. These data are now included in the revised manuscript (Fig. EV2D-E, line 175).

Minor issues:

1. The abstract needs some proofreading. e.g.: " Deletion mutations in the translation termination factor, HBS1L, result in..." should not have commas around HBS1L
" Mechanistically, we foud that..." spelling/typo

In the intro " Loss of ATF4 has been linked to a plethora of developmental abnormalities in the skeletal system, hematopoiesis, adipose and liver tissues"
Hematopoiesis should be "hematopoietic system" because "hematopoiesis" is not a tissue.
>**We thank the reviewer for catching these errors and have corrected all the above issues.**

2. Fig 1A - In the overlay image is the GFP[nls] shown green or blue? Because it looks green in the image but blue in the labeling above the image. Also, I can clearly see the reduction in the 4E-BP reporter in the single-channel images, but it is difficult to see in the overlay - can the purple be made more clear? Or pick another color?
>**GFP^{NLS} is indeed in cyan (blue). The overlay image also contains DAPI in yellow and the 4E-BP reporter in magenta. When all three signals are present, the nuclei present as white (as seen in the $LacZ^{RNAi}$ image) but reduction in the reporter (as seen in the $Hbs1^{RNAi}$ image) presents as green (GFP in cyan+DAPI in yellow). We appreciate that this may be confusing for readers since GFP is commonly represented in green, but we would like to be consistent with the CMYK labeling that is color-blind reader friendly. To improve legibility, we have added this explanation to the figure legend.**

3. Fig 2C - is the 'control reporter' a reporter with the wildtype ATF4 5'UTR as shown in Fig 2D ? If so, it would be helpful to label the reporters in the schematic in Fig 2C as "control" and "mutant" to make this more clear. (In the Results text it's being referred to as "ATF4 5'-GFP reporter" instead of a control reporter.) This is particularly confusing because its levels do not increase in response to tunicamycin. If this is due to perdurance of the GFP, it would be worth replacing the GFP with either a destabilized GFP or luciferase in order to get a reporter that faithfully reproduces the regulation seen with endogenous ATF4.
>The control reporter is the wildtype ATF4 5'UTR reporter. We agree that our current labeling is misleading and have corrected the text and the figures to consistently refer to this as 'wildtype'. We have also labeled the schematic in Fig. 2D per the reviewer's suggestion.
The reviewer is correct that we likely do not see an induction in GFP with the wildtype reporter in Fig. 2C because of the perdurance of GFP. Per the reviewer's suggestion, we obtained similar reporters with renilla luciferase (ATF4 5' leader-luciferase, PMID 32938922) and repeated these experiments. Consistent with our data in Fig. 2C, we find that knocking down either HBS1L or Pelo results in diminished induction of the luciferase. These data are included in the revised manuscript (Fig. EV2B-C, line 172).

4. The drop in global translation rates upon Pelo depletion specifically in the Tunicamycin condition is interesting because it suggests that Pelo activity is more required when eIF2-alpha is phosphorylated. Perhaps it is worth discussing this?
>We agree with the reviewer that this is indeed an intriguing finding. Our laboratory is actively investigating the hypothesis that HBS1L-Pelo activity is more relevant under conditions of ISR activation (when eIF2α is phosphorylated) rather than under 'homeostasis'. This is supported by its role in regulating the translation of ATF4, which is a stress-responsive transcript. In the original manuscript, we had elected to not discuss the difference in new protein synthesis with Pelo knockdown since the data were not statistically significant. However, given the broader trend indicating a role for HBS1L-Pelo during stress, we have now acknowledged such a possible role in the discussion section of the revised manuscript (line 378).

5. The sentence " our ERG analyses revealed that Gcm>LacZ expression resulted in on- and off-transient defects in Hbs11/Hbs148 animals when compared to control w1118 animals (Fig. 5E-

G)" is a bit confusing because, if I understand correctly, it's not the LacZ expression that results in the defect, but the Hbs1 mutation.
>We have edited this sentence for clarity (line 303).

Referee #2:

The paper by Querry et al. describes how the loss of the ribosome rescue factors Hbs1 or Pelo in Drosophila result in the loss of ATF4. The same appears to be true in human cell lines using knockdown of HBS1L and PELO. The authors examine retinal function in Drosophila models with knockdown or mutations in Hbs1, Pelo, and Atf4/crc, given the optic phenotypes of patients with mutations in these genes. They find a variety of ERG problems that primarily point to neurons in the medulla, although photoreceptor degeneration/dysfunction is also involved in some genotypes and at later ages. Importantly, the ERG phenotype of Hbs1 mutants can largely be rescued by expression of Atf4 without its normal regulatory 5' end. Overall, the paper is well written, the data are clear and compelling, and the results are well quantified. A few additions would further strengthen the manuscript.
We thank the reviewer for their positive view of our work and their feedback, which we believe have strengthened our conclusions.

1. The rescue of Hbs1 mutant ERGs with expression of Atf4 is interesting, but also unexplained. It is possible that unregulated expression of Atf4 is compensating for or otherwise circumventing the loss Hbs1. The extent to which the loss of Atf4 is central to the ERG phenotypes of Pelo and Hbs1 knockdown (3C, 4A, 5A) should be tested by knockdown or conditional knockdown of Atf4 in those backgrounds to see if there is an additional effect.
>We agree with the reviewer that the partial rescue of ERG defects seen with ATF4 expression in *Hbs1* mutants could be correlative and not causative. Per the reviewer's suggestion, we did a double knockdown of Pelo or Hbs1 along with ATF4 in lamina neurons with Gcm-GAL4 and measured ERG. However, in our double knockdown animals (*Hbs1*^{RNAi}+*ATF4*^{RNAi} and *pelo*^{RNAi}+*ATF4*^{RNAi}) we only saw a mild reduction in on-transients with no significant effects on off-transients or receptor potential in comparison to control (data below). We believe this may be due to insufficient knockdown of either gene due to dilution of GAL4 activity across two UAS-RNAi elements. We have previously reported such dilution to be true for *ATF4*^{RNAi} in a different tissue where double knockdowns diminished loss-of-function phenotypes (Grmai et al, Cell Reports, 2024). We also attempted testing the premise of whether double knockdown exacerbates defective ERGs by trying to knockdown *Hbs1* or *pelo* in the *ATF4* mutant background, but were unable to recover any recombinants of *Gcm-GAL4* with the *ATF4* mutant allele. Unfortunately, these results render this experiment inconclusive and we thus elected to not include it in the revised manuscript.
In light of us not being able to address this issue, we have caveated our conclusions around these experiments in the text to leave open the possibility of other Hbs1-Pelo targets being involved (line 306).

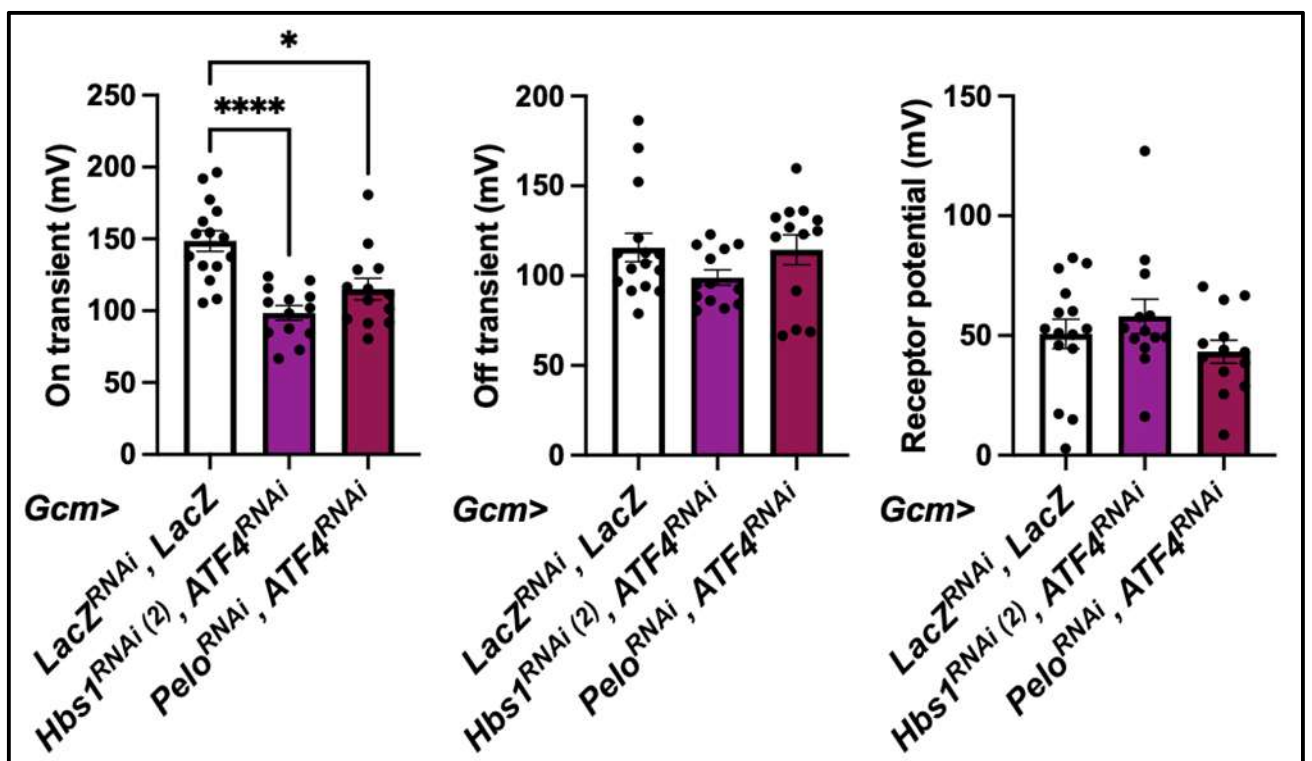

Fig. Quantification of ERG parameters from 3-5 day old animals where the lamina driver (Gcm-GAL4) is used to express knockdown two control UAS elements (LacZ^{RNAi}, LacZ) or doubly knockdown ATF4 with either Hbs1 or Pelo. Statistical comparisons were made using the Kruskal-Wallist test for multiple comparisons.

Minor points:
1. In results of figure 5, the authors use the term "ATF4 expression." As a general rule, genes are expressed and proteins are at "levels" of "abundance". It seems the authors are referring to ATF4 protein levels, not gene/mRNA expression levels, so "expression" may not be the best descriptor.
>**We edited this to "ATF4 protein" and have corrected other similar errors across the manuscript to the best of our ability.**

2. In figures 1 and S1, I am unfamiliar with fat body anatomy, can the authors please provide a brief description.
>In the wandering third-instar larval stage, the *Drosophila* fat body forms a large, sheet-like organ composed of polyploid adipocyte-like cells that line the inner body cavity beneath the cuticle. These cells are arranged in loosely organized lobes that surround the gut and other organs, creating a metabolically active tissue that functions analogously to mammalian adipose tissue and liver. The fat body stores lipids and glycogen and serves as a major endocrine and metabolic hub during the larval-to-pupal transition. We have also included a brief descriptor in the revised manuscript (line 130).

3. The Discussion section gets a bit speculative on connections between Drosophila retinal phenotypes and the human disease. I suggest tightening up those comparisons and spending additional time on how ATF4, as a transcription factor, may be manifesting these effects. Are there known ATF4 1. genes that could contribute, etc. Similarly, is chronically high ATF4 anticipated to be deleterious?
>We appreciate the reviewer's viewpoint that our discussion comparing the human defects with the *Drosophila* model are speculative in nature. However, our current understanding of the etiology of the human disease is very limited, and progress on this front is likely to be slow in other models of the disease given limited genetic tool kits to probe cell-type specific requirements for Hbs1. Given these, we believe it to be important to discuss how our findings in the fly model can inform the human disease in detail.
We agree that the manuscript lacked discussion on how ATF4 may be mediating lamina neuron development. Previous studies link loss of ATF4 in accelerated retinal degeneration (PMIDs 34919148) and chronically high ATF4 to also accelerate retinal degeneration (PMID27144303). This is largely attributed to ATF4 activity in photoreceptors, which are very secretory in nature and known to be reliant on ER stress response pathways for their function. However, specific targets of ATF4 that contribute to these phenotypes remain understudied and no speculations have been made to the best of our knowledge. Further, our study suggests a role for ATF4 in lamina neurons, which are not typically considered to have a high secretory load, making speculation of ATF4 targets even less reliable. We have included these ideas in the discussion leaving room for future studies to resolve this (line 438, 445).

Referee #3:
This manuscript reports a requirement for the ribosome rescue factors Hbs1 and Pelota in development of visual system neurons. It is first reported that loss of either of these rescue factors, which function together as a heterodimer, reduces integrated stress response (ISR) transcription in Drosophila cells. The ISR is driven by the translationally-regulated transcription factor Atf4, and depletion of Hbs1 or Pelo also reduces Atf4 protein levels in human cells. Building on the published observation that human disease mutations and mouse genetic models lacking Hbs1 show visual defects, the effects of these mutants are tested in the Drosophila visual system.

Genetic disruption of Hbs1, Pelo, or Atf4 greatly disrupt the transient and steady-state electrical responses to light in Drosophila photoreceptors. These altered responses are partially reproduced by cell type-specific depletion of Hbs1, Pelo, or Atf4 in lamina neurons downstream of photoreceptors. Furthermore, Atf4 overexpression in these lamina neurons can partially suppress Hbs1 depletion. No dramatic changes in cell or tissue organization were seen in Hbs1-depleted lamina, however.

This manuscript describes interesting consequences of Hbs1 depletion in Drosophila. From a molecular perspective, the role for these factors in Atf4 expression is interesting. Likewise, the impact of these pathways on the visual system is notable. However, the connection between these observations is tenuous and neither is analyzed in depth. Furthermore, in both cases, the lack of deeper analysis raises the possibility that indirect and uninformative effects could explain the observed phenomena. My sense is that reasonable revisions could not address these fundamental concerns with the current manuscript. For these reasons, I recommend against publication, with my specific concerns enumerated below.

We appreciate the reviewer's concerns surrounding mechanistic depth and hope that the additional experiments and responses below serve to remedy many, if not all, of their issues and persuade them to reconsider their assessment.

1. Loss of Hbs1 and Pelo could indirectly activate the ISR. Loss of HBS1L causes global shifts in protein synthesis and altered mTOR signaling (O'Connell et al., 2019), and similar effects could likewise alter ISR activation. Further mechanistic analysis, such as analysis of eIF2α phosphorylation and its regulation, could be valuable for addressing this broad concern.

>We generally agree with the reviewer that there may be other mechanisms at play in HBS1L-Pelo-mediated regulation of ATF4. As the reviewer pointed out, HBS1L-Pelo may impact reinitiation on the 5'leader by altering ISR activation, which would manifest as differences in phosphorylation of eIF2α. To test this possibility, we performed western blotting analysis in lysates where we depleted HBS1L/Pelo. We found that the basal levels of P-eIF2α in 293T cells was high (Fig. EV3B), thus we did not measure a statistically significant induction with our Tunicamycin-treatment paradigm (Fig. EV3C). However, unlike with ATF4 protein whose induction with Tunicamycin is diminished in HBS1L/Pelo RNAi samples, we see no changes in P-eIF2α with HBS1L/Pelo RNAi. These results rule out the possibility that HBS1L/Pelo may act on upstream ISR effects. These results are now included in the manuscript (Fig. EV3B-C, line 194). While there may still be contributory effects of mTOR and other translational control pathways, these data strongly suggest that the effects of HBS1L-Pelo are due to regulation of translation reinitiation in the ATF4 5' leader.

2. Related to this point, the lack of regulated translation limits the value of 5′ UTR reporter used in Figure 2.

3. It is hard to reconcile the apparent band intensities in Figure 2A with the quantitation in Figure 2C. In Figure 2A, the "GFP" blot has notably faint bands for HBS1L Tu- and both Pelo lanes relative to the others, and quite consistent "Tub" control. The quantitation in Figure 2C shows no difference in HBS1L +/- Tunicamycin, however, and only a modest ~2-fold reduction in the Pelo samples.

>Similar concerns were also raised by referee 1 (point 3), who suggested that we test a different 5' leader reporter such as luciferase and also whether our proposed mechanism of HBS1L-Pelo is evolutionarily conserved in *Drosophila*. Per their suggestion, we tried these additional reporters and observed that knockdown of either HBS1L or Pelo resulted in reduced ATF4 5'-luciferase levels (Fig. EV2B-C) and reduced *Drosophila* ATF4 5'-DsRed levels (Fig. EV2D-E). While these results strengthen our conclusion that the effects of HBS1-Pelo depletion are 5' leader dependent, we agree with Referee 3 that we see at most

a two-fold reduction in reporter levels with our loss-of-function analyses. This 'modest' defect is why are we argue that HBS1L-Pelo regulates efficiency of reinitiation at the ATF4 5'-leader, instead of presenting them as 'required' factors.

4. It is written that, "knockdown of Pelo resulted in a small but statistically significant reduction in the mutant reporter." Why is this not shown on the graph with significance marks?
>We thank the reviewer for catching this error in figure preparation. We have now corrected this in the revised Fig. 2F and added error bars.

5. In Figure 3C, a pelo; Hbs1 double heterozygote is analyzed along with a biallelic Hbs1 mutant and an Atf4 heterozygote. Was any analysis performed of single heterozygotes for either pelo or Hbs1?
>We had also performed ERG analyses on heterozygous *Hbs1* and *pelo* mutant animals but had not included the data since these animals did not show any ERG defects. However, we appreciate that these data maybe helpful in delineating the contribution of the individual mutant alleles and have thus included these in the revised manuscript (Fig EV4A-C, line 237).

6. Lamina neuron depletion of Hbs1 (Figure 4) has a weaker and age-dependent effect relative to the global effects of Hbs1 depletion. Were photoreceptor-specific depletions tested?
>We thank the reviewer for raising the question of whether Hbs1 is required in photoreceptors. To test this, we employed the photoreceptor-specific driver, *Rh1-GAL4* to deplete Hbs1 throughout development and performed ERG on young adults. We did not see an appreciable change in any of the ERG parameters (Fig. EV4D-F). However, as with the glial driver, *repo-GAL4* (Fig. EV4G-I), we saw a small but statistically insignificant decrease in receptor potential with *Rh1-GAL4*, which may be indicative of the onset of age-related effects in both glia and photoreceptors. We have included these data in the revised manuscript (Fig. EV4D-F, line 257) and discussed the possible age-related implications as well (line 453).

7. More broadly, what sensitizes laminar neurons in particular to this defect in protein synthesis? Photoreceptor cells have highly specialized physiology and consequently display some particular sensitivities to protein folding and processing. Other highly specialized secretory cells depend particularly on the ISR. Why these laminar neurons?
>The reviewer raises an excellent big-picture question and is similar to a comment made by referee 2 (minor point 3) on potential transcriptional targets of ATF4 that are required in lamina neurons. There is no precedent in literature for lamina neurons to be reliant on ISR signaling (whether to manage ER stress or nutrient challenges) or other pathways that enforce selective protein synthesis. Vacuolization defects such as those we observe in in *Hbs1* mutants (Fig. 6) have been previously reported due to hyperwrapping of glia (e.g. PMID 9295388) or trafficking defects in photoreceptors (e.g. PMID 25909451). However, not much is known about lamina-autonomous processes that result in vacuolization defects and this is an avenue of research actively being pursued in our laboratories. Unfortunately, the set of experiments to determine the cell biological basis for why lamina neurons are particularly sensitive to selective mRNA translation is rather extensive. Thus, we respectfully present that this question is beyond the scope of this manuscript.

1st Revision - Editorial Decision 29th Apr 2026

Dear Dr. Vasudevan,

Thank you for the submission of your revised manuscript to our editorial offices. I have now received the reports from the three referees that were asked to re-evaluate the study, you will find below. As you will see, the referees now support the publication of the study in EMBO reports. Referee #3 has a suggestion and a final comment, I ask you to address in a final revised manuscript. Please also provide a final p-b-p-response to these points and to the editorial requests below.

Editorial requests:

- I would suggest this slightly different title:
Visual system function requires translational regulation of ATF4 by Hbs1-Pelo

- Please order the manuscript sections like this, using only these names:
Title page - Abstract - Keywords - Introduction - Results - Discussion - Methods - Data availability section - Acknowledgements - Disclosure and Competing Interests Statement - References - Figure legends - Expanded View Figure legends

Please merge the funding information with the Acknowledgements.

- We now use CRediT to specify the contributions of each author in the journal submission system. CRediT replaces the author contribution section. Please use the free text box to provide more detailed descriptions and do NOT provide your final manuscript text file with an author contributions section. See also our guide to authors (section 'Author contributions'): https://link.springer.com/journal/44319/submission-guidelines#cms-Revised-submissions

- We updated our journal's competing interests policy in January 2022 and request authors to consider both actual and perceived competing interests. Please name this section 'Disclosure and Competing Interests Statement' and put it after the Acknowledgements section. See: https://link.springer.com/partners/embo-press/editorial-policies#Competing%20interest%20disclosures

- Please check again that the number "n" for how many independent experiments were performed, their nature (biological versus technical replicates), the bars and error bars (e.g. SEM, SD) and the test used to calculate p-values is indicated in the respective figure legends (main and EV figures). Please also check that all the p-values are explained in the legend, and that these fit to those shown in the figure. Please provide statistical testing where applicable. Please avoid the phrase 'independent experiment' but clearly state if these were biological or technical replicates. Please also indicate (e.g. with n.s.) if testing was performed, but the differences are not significant. In case n=2, please show the data as separate datapoints without error bars and statistics. See also:

https://link.springer.com/journal/44319/submission-guidelines#cms-Figure-and-data-presentation

If n<5, please show single datapoints for diagrams. Presently, several diagrams lack statistics or the 'n.s.' (that seems not to be indicated in any diagram). Please provide proper and complete statistical testing for all diagrams shown. Moreover:
- Please note that the legend for figure EV5B is missing in the manuscript text. This needs to be rectified.
- Please define the annotated p values ****/***/**/* as well as provide the exact p-values for the same in the legend of figure 3E-G; 4C-E; 5B, C, F, G as appropriate.
- Please note that the exact p values are not provided in the legends of figures 1B, D, F; 2B, C, F, G; S2C, E.

- Panel 6D is not labelled in the figure file. Please check.

- Please use our reference format:
https://link.springer.com/journal/44319/submission-guidelines#cms-Reference-guidelines

Please use et al after 10 author names; DOIs should only be used for preprints and datasets that have not been published yet.

- Please make sure that all the funding information is also entered into the online submission system and that it is complete and similar to the one in the acknowledgement section of the manuscript text file. Presently, these grants are missing from the submission system: R35GM150516, NIHR00EY029013, 225986. Please check.

- Please make sure that each figure panel is called out separately and that the panels are called out sequentially. Presently a panel 6F called out in the text, but the panel is missing in the figure. Separate call outs for the two panels of Fig. EV5 seem missing. Please check.

- All Materials and Methods need to be described in the main text using our 'Structured Methods' format, which is required for all research articles. According to this format, the Methods section should include a Reagents and Tools Table (listing key

reagents, experimental models, software, and relevant equipment and including their sources and relevant identifiers), uploaded as separate file, and a Methods section in which we encourage the authors to describe their methods using a step-by-step protocol format with bullet points, to facilitate the adoption of the methodologies across labs. More information on how to adhere to this format as well as downloadable templates (.doc) for the Reagents and Tools Table can be found in our author guidelines (section 'Structured Methods'):

https://link.springer.com/journal/44319/submission-guidelines#cms-Manuscript-organisation-and-formatting

Please add primer or antibody information directly to the Reagents and Tools table.

- Please name the two tables in the manuscript 'Table 1' and 'Table 2'. These are main tables. Moreover, please place these tables in between the main and EV figure legends.

In addition, I would need from you uploaded separately:
- a short, two-sentence summary of the manuscript (not more than 35 words).
- two to four short (!) bullet points highlighting the key findings of your study (two lines each).
- a schematic summary figure as separate file that provides a sketch of the major findings (not a data image) in jpeg or tiff format (with the exact width of 550 pixels and the exact height of 300 pixels) that can be used as a visual synopsis on our website.

I look forward to seeing the further revised version of your manuscript when it is ready. Please let me know if you have questions regarding the revision.

Best,

Achim Breiling
Senior Editor
EMBO Reports

Referee #1:

The authors have nicely addressed all the concerns raised in my original review.

Referee #2:

The paper describes the role of Pelo and Hbs1 in the translation of ATF4. As ATF4 is a key player in the integrated stress response and its levels are controlled largely by translation, a more thorough understanding of this mechanism is important in the field.

The authors have addressed my earlier concerns. I appreciate the effort to look at the loss of ATF4 in the background of Hbs1 or Pelo and I accept the explanation provided in the response to reviews that these experiments were not feasible for technical reasons. My other concerns were minor, and the authors were responsive to the comments of the other reviewers.

Referee #3:

Revisions have addressed my key concerns with the original manuscript. The new 5′ UTR reporter, currently in EV2B-C, shows that HBS1L and PELO depletion alter the distinctive regulation of ATF4. These data provide stronger support for the claim that HBS1L and PELO are affecting ATF4 directly, and I wonder whether it would be more useful to display this as a main figure.

The new mutant electrophysiology-showing that photoreceptor-specific Hbs1 RNAi does not show significant changes, nor do individual pelo and Hbs1 heterozygotes-is valuable for showing the specificity of the effects.

REFEREE RESPONSE

We thank the referees for their fair and positive assessment of our work. Referees 1 and 2 had no addressable comments and we address Referee 3's request below.

Referee #3:

Revisions have addressed my key concerns with the original manuscript. The new 5′ UTR reporter, currently in EV2B-C, shows that HBS1L and PELO depletion alter the distinctive regulation of ATF4. These data provide stronger support for the claim that HBS1L and PELO are affecting ATF4 directly, and I wonder whether it would be more useful to display this as a main figure.

>**We appreciate the suggestion from the referee. However, our view is that the mechanistic understanding for how HBS1L-Pelo affect ATF4 comes from the cell culture-based reporter analyses. The *Drosophila* reporter only serves as a validation of the findings and thus we believe it is best relegated to the supplement.**

EMBO EDITOR REQUEST CHECKLIST

1. I would suggest this slightly different title:
   Visual system function requires translational regulation of ATF4 by Hbs1-Pelo
   >We appreciate the more succinct title suggestion and have altered it accordingly.

2. Please order the manuscript sections like this, using only these names:
   Title page - Abstract - Keywords - Introduction - Results - Discussion - Methods - Data availability section - Acknowledgements - Disclosure and Competing Interests Statement - References - Figure legends - Expanded View Figure legends
   >We have arranged the sections per this order and changed titles accordingly.

3. Please merge the funding information with the Acknowledgements.
   - We now use CRediT to specify the contributions of each author in the journal submission system. CRediT replaces the author contribution section. Please use the free text box to provide more detailed descriptions and do NOT provide your final manuscript text file with an author contributions section. See also our guide to authors (section 'Author contributions'):
   https://link.springer.com/journal/44319/submission-guidelines#cms-Revised-submissions
   >We have made these changes.

4. We updated our journal's competing interests policy in January 2022 and request authors to consider both actual and perceived competing interests. Please name this section 'Disclosure and Competing Interests Statement' and put it after the Acknowledgements section. See: https://link.springer.com/partners/embo-press/editorial-policies#Competing%20interest%20disclosures ✅
   >We have made these changes.

5. Please check again that the number "n" for how many independent experiments were performed, their nature (biological versus technical replicates), the bars and error bars (e.g. SEM, SD) and the test used to calculate p-values is indicated in the respective figure legends (main and EV figures). Please also check that all the p-values are explained in the legend, and that these fit to those shown in the figure. Please provide statistical testing where applicable. Please avoid the phrase 'independent experiment' but clearly state if these were biological or technical replicates. Please also indicate (e.g.

with n.s.) if testing was performed, but the differences are not significant. In case n=2, please show the data as separate datapoints without error bars and statistics. ✅
See also: https://link.springer.com/journal/44319/submission-guidelines#cms-Figure-and-data-presentation
If n<5, please show single datapoints for diagrams. Presently, several diagrams lack statistics or the 'n.s.' (that seems not to be indicated in any diagram). Please provide proper and complete statistical testing for all diagrams shown. Moreover:
- Please note that the legend for figure EV5B is missing in the manuscript text. This needs to be rectified.
- Please define the annotated p values ****/***/**/* as well as provide the exact p-values for the same in the legend of figure 3E-G; 4C-E; 5B, C, F, G as appropriate. ✅
- Please note that the exact p values are not provided in the legends of figures 1B, D, F; 2B, C, F, G; S2C, E.
- Panel 6D is not labelled in the figure file. Please check.
> Individual data points are now shown for all graphs. We listed 'n' and included details on biological vs technical replicates in all figure legends. We have updated graphs to include n.s. where we performed comparisons and included exact p-values in the figure legends. For image analyses where p<0.0001, the p-values are approximate due to the size of the data set and we specify this in the legend. We also include the legend of EV5B and ensured panel 6D is labeled.

6. Please use our reference format:
https://link.springer.com/journal/44319/submission-guidelines#cms-Reference-guidelines
Please use et al after 10 author names; DOIs should only be used for preprints and datasets that have not been published yet.
- Please make sure that all the funding information is also entered into the online submission system and that it is complete and similar to the one in the acknowledgement section of the manuscript text file. Presently, these grants are missing from the submission system: R35GM150516, NIHR00EY029013, 225986. Please check.
>We have updated the reference list to use the EMBO journal format.

7. Please make sure that all the funding information is also entered into the online submission system and that it is complete and similar to the one in the acknowledgement section of the manuscript text file. Presently, these grants are missing from the submission system: R35GM150516, NIHR00EY029013, 225986. Please check
>We have added these to the online submission system.

8. Please make sure that each figure panel is called out separately and that the panels are called out sequentially. Presently a panel 6F called out in the text, but the panel is missing in the figure. Separate call outs for the two panels of Fig. EV5 seem missing. Please check
>We have removed the erroneous reference to panel 6F and EV5.
Regarding calling out panels separately and sequentially: we have rearranged Fig. 2 to comply with this. However, for Figures 3, 4 and EV4, we are unable to do so. This is because the quantifications for the individual traces are consolidated (e.g. traces in 3A-D are consolidated in 3E-G, thus 3E-G needs to evoked prior to 3B), which we believe is the best way to represent these data.

9. All Materials and Methods need to be described in the main text using our 'Structured Methods' format, which is required for all research articles. According to this format, the

Methods section should include a Reagents and Tools Table (listing key reagents, experimental models, software, and relevant equipment and including their sources and relevant identifiers), uploaded as separate file, and a Methods section in which we encourage the authors to describe their methods using a step-by-step protocol format with bullet points, to facilitate the adoption of the methodologies across labs. More information on how to adhere to this format as well as downloadable templates (.doc) for the Reagents and Tools Table can be found in our author guidelines (section 'Structured Methods'):
https://link.springer.com/journal/44319/submission-guidelines#cms-Manuscript-organisation-and-formatting
Please add primer or antibody information directly to the Reagents and Tools table.
- Please name the two tables in the manuscript 'Table 1' and 'Table 2'. These are main tables. Moreover, please place these tables in between the main and EV figure legends.
>We have rewritten the methods using the appropriate format and generated a Reagents and Tools table as well.

10. In addition, I would need from you uploaded separately:
    - a short, two-sentence summary of the manuscript (not more than 35 words).
    >The following summary has been uploaded separately.

    The ribosome recycling complex Hbs1-Pelo regulates expression the stress response transcription factor ATF4 in visual system development and function. In Drosophila and human cells Hbs1-Pelo regulate ATF4 translation via its 5' leader.

11. two to four short (!) bullet points highlighting the key findings of your study (two lines each).
    >Please see bullet points below.
    - The ribosome recycling complex Hbs1-Pelo regulates ATF4 translation in *Drosophila* and human cells
    - Hbs1 and Pelo are required for efficient translation termination at upstream ORFs in the ATF4 5' leader
    - In *Drosophila*, Hbs1-Pelo is required in lamina neurons, which interface with photoreceptors, for proper phototransduction
    - Restoring ATF4 in lamina neurons partially rescues phototransduction defects seen in *Hbs1* mutants

12. a schematic summary figure as separate file that provides a sketch of the major findings (not a data image) in jpeg or tiff format (with the exact width of 550 pixels and the exact height of 300 pixels) that can be used as a visual synopsis on our website.
    >We have uploaded a summary figure.

2nd Revision - Editorial Decision 9th Jul 2026

Dr. Deepika Vasudevan
University of Pittsburgh School of Medicine
Cell Biology
United States

Dear Dr. Vasudevan,

Thank you for the submission of your further revised manuscript to our editorial offices. I now went through this and your final p-b-p-response and consider the remaining points of the referee and the editorial requests as adequately addressed.

I am thus very pleased to accept your manuscript for publication in the next available issue of EMBO reports. Thank you for your contribution to our journal.

Your manuscript will be processed for publication by EMBO Press. It will be copy edited and you will receive page proofs prior to publication. Please note that you will be contacted by Springer Nature Author Services to complete licensing and payment information.

You may qualify for financial assistance for your publication charges - either via a Springer Nature fully open access agreement or an EMBO initiative. Check your eligibility: https://link.springer.com/journal/44319/how-to-publish-with-us

Should you be planning a Press Release on your article, please notify the production team via this form in order to coordinate publication and release dates: https://forms.cloud.microsoft/e/eH4A8JVP6g

If you have any questions, please do not hesitate to contact the Editorial Office. Thank you for your contribution to EMBO Reports.

Yours sincerely,

Achim Breiling
Senior Editor
EMBO Reports

>>> Please note that it is EMBO Reports policy for the transcript of the editorial process (containing referee reports and your response letter) to be published as an online supplement to each paper. If you do NOT want this, you will need to inform the Editorial Office via email immediately. More information is available here: https://link.springer.com/partners/embo-press/editorial-policies#Peer%20review

EMBO Press Author Checklist

Corresponding Author Name: Deepika Vasudevan
Journal Submitted to: EMBO Reports
Manuscript Number: EMBOR-2025-62980V2

USEFUL LINKS FOR COMPLETING THIS FORM
The EMBO Journal - Author Guidelines
EMBO Reports - Author Guidelines
Molecular Systems Biology - Author Guidelines
EMBO Molecular Medicine - Author Guidelines

Reporting Checklist for Life Science Articles (updated January
This checklist is adapted from Materials Design Analysis Reporting (MDAR) Checklist for Authors. MDAR establishes a minimum set of requirements in transparent reporting in the life sciences (see Statement of Task: 10.31222/osf.io/9sm4x). Please follow the journal's guidelines in preparing your
Please note that a copy of this checklist will be published alongside your article.

Abridged guidelines for figures
1. Data
The data shown in figures should satisfy the following conditions:
- the data were obtained and processed according to the field's best practice and are presented to reflect the results of the experiments in an accurate and unbiased manner.
- ideally, figure panels should include only measurements that are directly comparable to each other and obtained with the same assay.
- plots include clearly labeled error bars for independent experiments and sample sizes. Unless justified, error bars should not be shown for technical
- if n<5, the individual data points from each experiment should be plotted. Any statistical test employed should be justified.
- Source Data should be included to report the data underlying figures according to the guidelines set out in the authorship guidelines on Data

2. Captions
Each figure caption should contain the following information, for each panel where they are relevant:
- a specification of the experimental system investigated (eg cell line, species name).
- the assay(s) and method(s) used to carry out the reported observations and measurements.
- an explicit mention of the biological and chemical entity(ies) that are being measured.
- an explicit mention of the biological and chemical entity(ies) that are altered/varied/perturbed in a controlled manner.
- the exact sample size (n) for each experimental group/condition, given as a number, not a range;
- a description of the sample collection allowing the reader to understand whether the samples represent technical or biological replicates (including how many animals, litters, cultures, etc.).
- a statement of how many times the experiment shown was independently replicated in the laboratory.
- definitions of statistical methods and measures:
  - common tests, such as t-test (please specify whether paired vs. unpaired), simple χ2 tests, Wilcoxon and Mann-Whitney tests, can be unambiguously identified by name only, but more complex techniques should be described in the methods section;
  - are tests one-sided or two-sided?
  - are there adjustments for multiple comparisons?
  - exact statistical test results, e.g., P values = x but not P values < x;
  - definition of 'center values' as median or average;
  - definition of error bars as s.d. or s.e.m.

Please complete ALL of the questions below.
Select "Not Applicable" only when the requested information is not relevant for your study.

Materials

Newly Created Materials	Information included in the manuscript?	In which section is the information available? (Reagents and Tools Table, Materials and Methods, Figures, Data Availability Section)
New materials and reagents need to be available; do any restrictions apply?	Yes	

Antibodies	Information included in the manuscript?	In which section is the information available? (Reagents and Tools Table, Materials and Methods, Figures, Data Availability Section)
For **antibodies** provide the following information:<br>- Commercial antibodies: RRID (if possible) or supplier name, catalogue number and or/clone number<br>- Non-commercial: RRID or citation	Yes	

DNA and RNA sequences	Information included in the manuscript?	In which section is the information available? (Reagents and Tools Table, Materials and Methods, Figures, Data Availability Section)
**Short novel DNA or RNA including primers, probes**: provide the sequences.	Yes	

Cell materials	Information included in the manuscript?	In which section is the information available? (Reagents and Tools Table, Materials and Methods, Figures, Data Availability Section)
**Cell lines:** Provide species information, strain. Provide accession number in repository **OR** supplier name, catalog number, clone number, and/**OR** RRID.	Yes	
**Primary cultures:** Provide species, strain, sex of origin, genetic modification status.	Not Applicable	
Report if the cell lines were recently **authenticated** (e.g., by STR profiling) and tested for mycoplasma contamination.	Not Applicable	

Experimental animals	Information included in the manuscript?	In which section is the information available? (Reagents and Tools Table, Materials and Methods, Figures, Data Availability Section)
**Laboratory animals or Model organisms:** Provide species, strain, sex, age, genetic modification status. Provide accession number in repository **OR** supplier name, catalog number, clone number, **OR** RRID.	Yes	
**Animal observed in or captured from the field:** Provide species, sex, and age where possible.	Not Applicable	
Please detail **housing and husbandry conditions**.	Yes	

Plants and microbes	Information included in the manuscript?	In which section is the information available? (Reagents and Tools Table, Materials and Methods, Figures, Data Availability Section)
**Plants:** provide species and strain, ecotype and cultivar where relevant, unique accession number if available, and source (including location for collected wild specimens).	Not Applicable	
**Microbes:** provide species and strain, unique accession number if available, and source.	Not Applicable	

Human research participants	Information included in the manuscript?	In which section is the information available? (Reagents and Tools Table, Materials and Methods, Figures, Data Availability Section)
If collected and within the bounds of privacy constraints report on age, sex and gender or ethnicity for all study participants.	Not Applicable	

Core facilities	Information included in the manuscript?	In which section is the information available? (Reagents and Tools Table, Materials and Methods, Figures, Data Availability Section)
If your work benefited from core facilities, was their service mentioned in the acknowledgments section?	Yes	

Design

Study protocol	Information included in the manuscript?	In which section is the information available? (Reagents and Tools Table, Materials and Methods, Figures, Data Availability Section)
If study protocol has been **pre-registered, provide DOI in the manuscript**. For clinical trials, provide the trial registration number **OR** cite DOI.	Not Applicable	
Report the **clinical trial registration number** (at ClinicalTrials.gov or equivalent), where applicable.	Not Applicable	

Laboratory protocol	Information included in the manuscript?	In which section is the information available? (Reagents and Tools Table, Materials and Methods, Figures, Data Availability Section)
Provide DOI OR other citation details if **external detailed step-by-step protocols** are available.	Not Applicable	

Experimental study design and statistics	Information included in the manuscript?	In which section is the information available? (Reagents and Tools Table, Materials and Methods, Figures, Data Availability Section)
Include a statement about **sample size** estimate even if no statistical methods were used.	Yes	
Were any steps taken to minimize the effects of subjective bias when allocating animals/samples to treatment (e.g. **randomization procedure**)? If yes, have they been described?	Not Applicable	
Include a statement about **blinding** even if no blinding was done.	Not Applicable	
Describe **inclusion/exclusion criteria** if samples or animals were excluded from the analysis. Were the criteria pre-established?<br><br>If sample or data points were omitted from analysis, report if this was due to attrition or intentional exclusion and provide justification.	Not Applicable	
For every figure, are **statistical tests** justified as appropriate? Do the data meet the assumptions of the tests (e.g., normal distribution)? Describe any methods used to assess it. Is there an estimate of variation within each group of data? Is the variance similar between the groups that are being statistically compared?	Yes	

Sample definition and in-laboratory replication	Information included in the manuscript?	In which section is the information available? (Reagents and Tools Table, Materials and Methods, Figures, Data Availability Section)
In the figure legends: state number of times the experiment was **replicated** in laboratory.	Yes	
In the figure legends: define whether data describe **technical or biological replicates**.	Yes	

Ethics

Ethics	Information included in the manuscript?	In which section is the information available? (Reagents and Tools Table, Materials and Methods, Figures, Data Availability Section)
Studies involving **human participants**: State details of **authority granting ethics approval** (IRB or equivalent committee(s), provide reference number for approval.	Not Applicable	
Studies involving **human participants**: Include a statement confirming that **informed consent** was obtained from all subjects and that the experiments conformed to the principles set out in the WMA Declaration of Helsinki and the Department of Health and Human Services Belmont Report.	Not Applicable	
Studies involving **human participants:** For publication of **patient photos**, include a statement confirming that consent to publish was obtained.	Not Applicable	
Studies involving experimental **animals**: State details of **authority granting ethics approval** (IRB or equivalent committee(s), provide reference number for approval. Include a statement of compliance with ethical regulations.	Not Applicable	
Studies involving **specimen and field samples:** State if relevant **permits** obtained, provide details of authority approving study; if none were required, explain why.	Not Applicable	

Dual Use Research of Concern (DURC)	Information included in the manuscript?	In which section is the information available? (Reagents and Tools Table, Materials and Methods, Figures, Data Availability Section)
Could your study fall under dual use research restrictions? Please check biosecurity documents and list of **select agents and toxins** (CDC): https://www.selectagents.gov/sat/list.htm	Not Applicable	
If you used a select agent, is the security level of the lab appropriate and reported in the manuscript?	Not Applicable	
If a study is subject to dual use research of concern regulations, is the name of the **authority granting approval and reference number** for the regulatory approval provided in the manuscript?	Not Applicable	

Reporting

The MDAR framework recommends adoption of discipline-specific guidelines, established and endorsed through community initiatives. Journals have their own policy about requiring specific guidelines and recommendations to complement MDAR.

Adherence to community standards	Information included in the manuscript?	In which section is the information available? (Reagents and Tools Table, Materials and Methods, Figures, Data Availability Section)
State if relevant guidelines or checklists (e.g., **ICMJE, MIBBI, ARRIVE, PRISMA**) have been followed or provided.	Not Applicable	
For **tumor marker prognostic studies,** we recommend that you follow the **REMARK** reporting guidelines (see link list at top right). See author guidelines, under 'Reporting Guidelines'. Please confirm you have followed these guidelines.	Not Applicable	
For **phase II and III randomized controlled trials**, please refer to the **CONSORT** flow diagram (see link list at top right) and submit the CONSORT checklist (see link list at top right) with your submission. See author guidelines, under 'Reporting Guidelines'. Please confirm you have submitted this list.	Not Applicable	

Data Availability

Data availability	Information included in the manuscript?	In which section is the information available? (Reagents and Tools Table, Materials and Methods, Figures, Data Availability Section)
Have **primary datasets** been deposited according to the journal's guidelines (see 'Data Deposition' section) and the respective accession numbers provided in the Data Availability Section?	Yes	
Were **human clinical and genomic datasets** deposited in a public access-controlled repository in accordance to ethical obligations to the patients and to the applicable consent agreement?	Not Applicable	
Are **computational models** that are central and integral to a study available without restrictions in a machine-readable form? Were the relevant accession numbers or links provided?	Not Applicable	
If publicly available data were reused, provide the respective **data citations in the reference list**.	Not Applicable	
