# Supplementary material for: Visual system function requires translational regulation of ATF4 by Hbs1-Pelo
Source: EMBO Rep. 2026 Jul 17;27(17):5121–42. doi: 10.1038/s44319-026-00882-6 (PMC13554125; doi:10.1038/s44319-026-00882-6)
Supplement: Supplementary file 2 [file 44319_2026_882_MOESM8_ESM.pdf]

Expanded View Figures

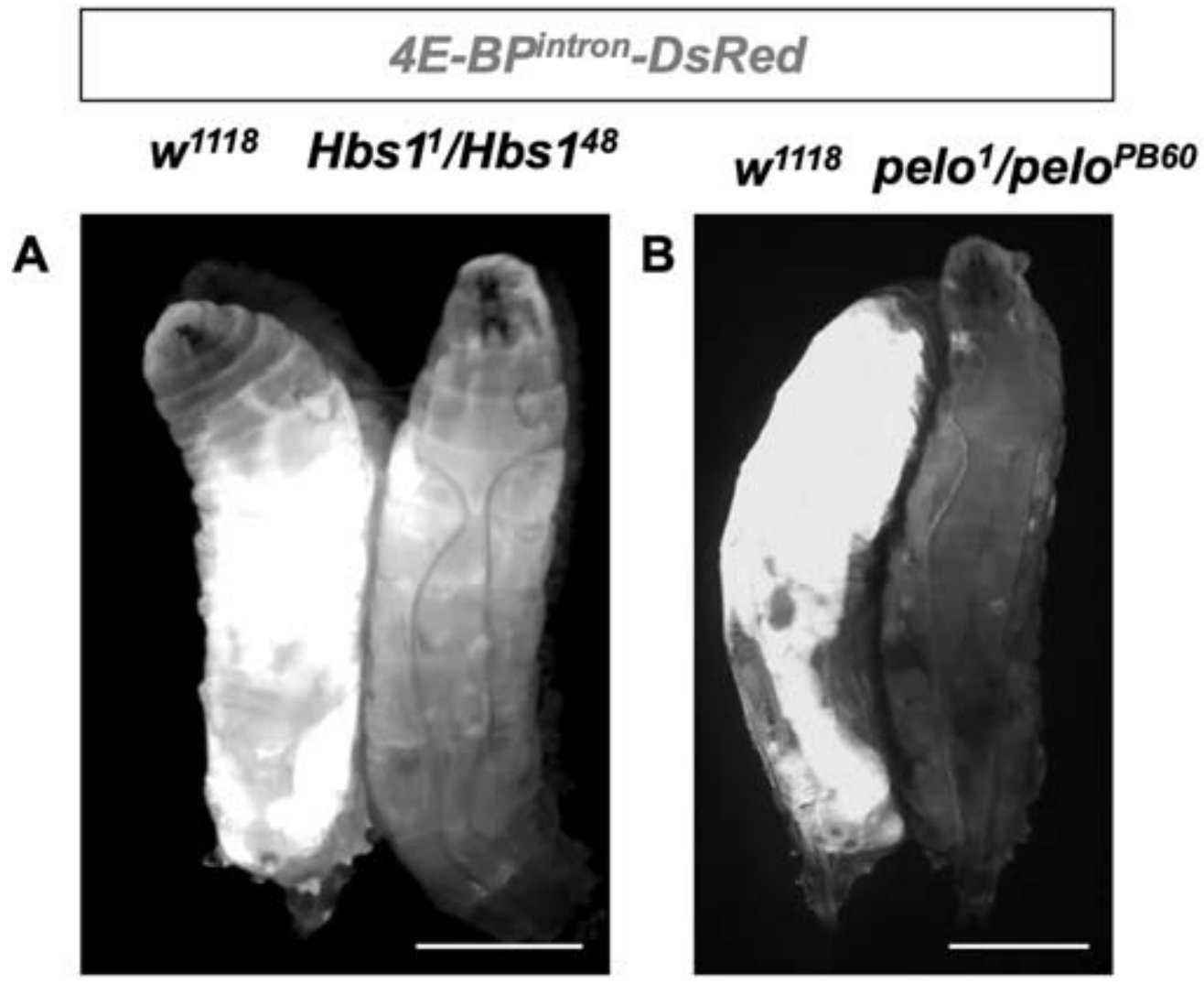

Figure EV1. Loss of *Hbs1* or *pelo* results in reduced ATF4 reporter expression.

Fluorescence images showing intensity of the ATF4 reporter, 4E-BP^{intron}-DsRed in live wandering third instar larva from wild type (w^{1118}), *Hbs1* (**A**) and *pelo* (**B**) mutants. Scale bars represent 1 µm.

© The Author(s)

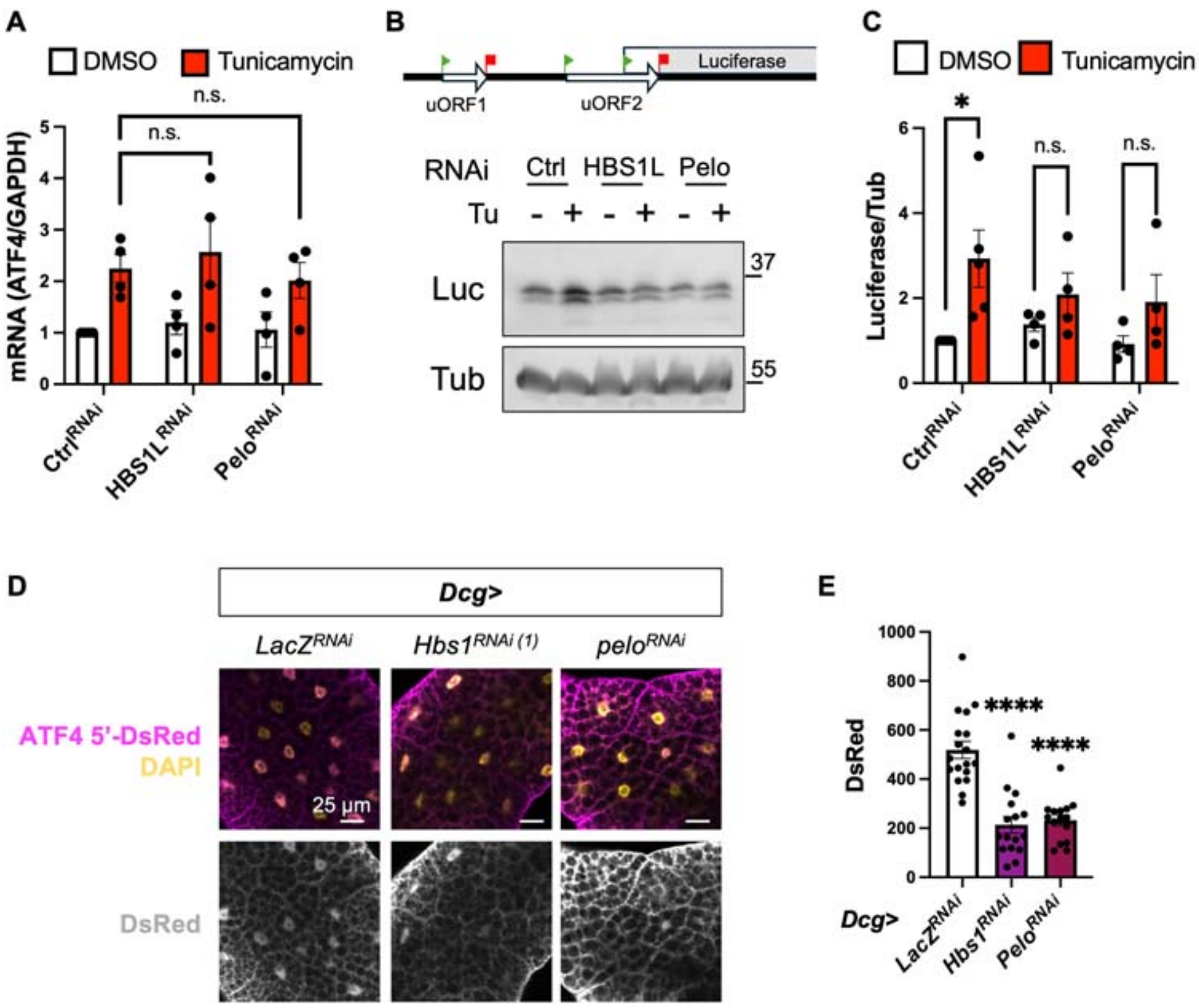

Figure EV2. HBS1L and Pelo act on the 5′ leader of human and *Drosophila* ATF4.

(**A**) q-RTPCR data measuring ATF4 mRNA normalized to house-keeping gene (GAPDH) in cells transfected with siRNA targeting HBS1L or Pelo and treated with 10μg/ml Tunicamycin to induce ER stress. Data represent mean of four biological replicates; error bars are standard error of mean. Statistical comparisons with Ctrl^{RNAi}-DMSO were performed using the Wilcoxon signed rank test for and are shown as floating asterisks when significant. Comparisons with Ctrl^{RNAi}-Tunicamycin were performed using the Friedman test with Dunn's correction. Other comparisons were performed using the Wilcoxon matched pairs signed rank test. (**B**) (Top) Schematic of the wild-type ATF4 5′ luciferase reporter and (bottom) reporter expression as measured by western blotting in cells treated as indicated. (**C**) Quantification of luciferase signal in (**B**) normalized to loading control (Tubulin). Data represent mean of at least four biological replicates; error bars are standard error of mean. Statistical comparisons were made using the Mann–Whitney test. *p*-values are as follows: columns 1 vs 2 = 0.03. (**D**) Representative confocal images of fat bodies expressing an ATF4 5′leader-DsRed reporter (magenta) in animals where *Hbs1* or *pelo* are depleted using *Dcg-GAL4*. DAPI marks nuclei in yellow. (**E**) Quantification of DsRed intensity from (**D**). Each data point represents a region of interest from a single animal, black bar represents mean of data from at least 5–7 biological replicates. Error bars represent standard error of mean. Statistical comparisons were made using Kruskal–Wallis test with Dunn's correction. *p*-values are approximate. Data information: ****$p < 0.0001$, ***$p < 0.001$, **$p < 0.01$, *$p < 0.05$, n.s. = not significant.

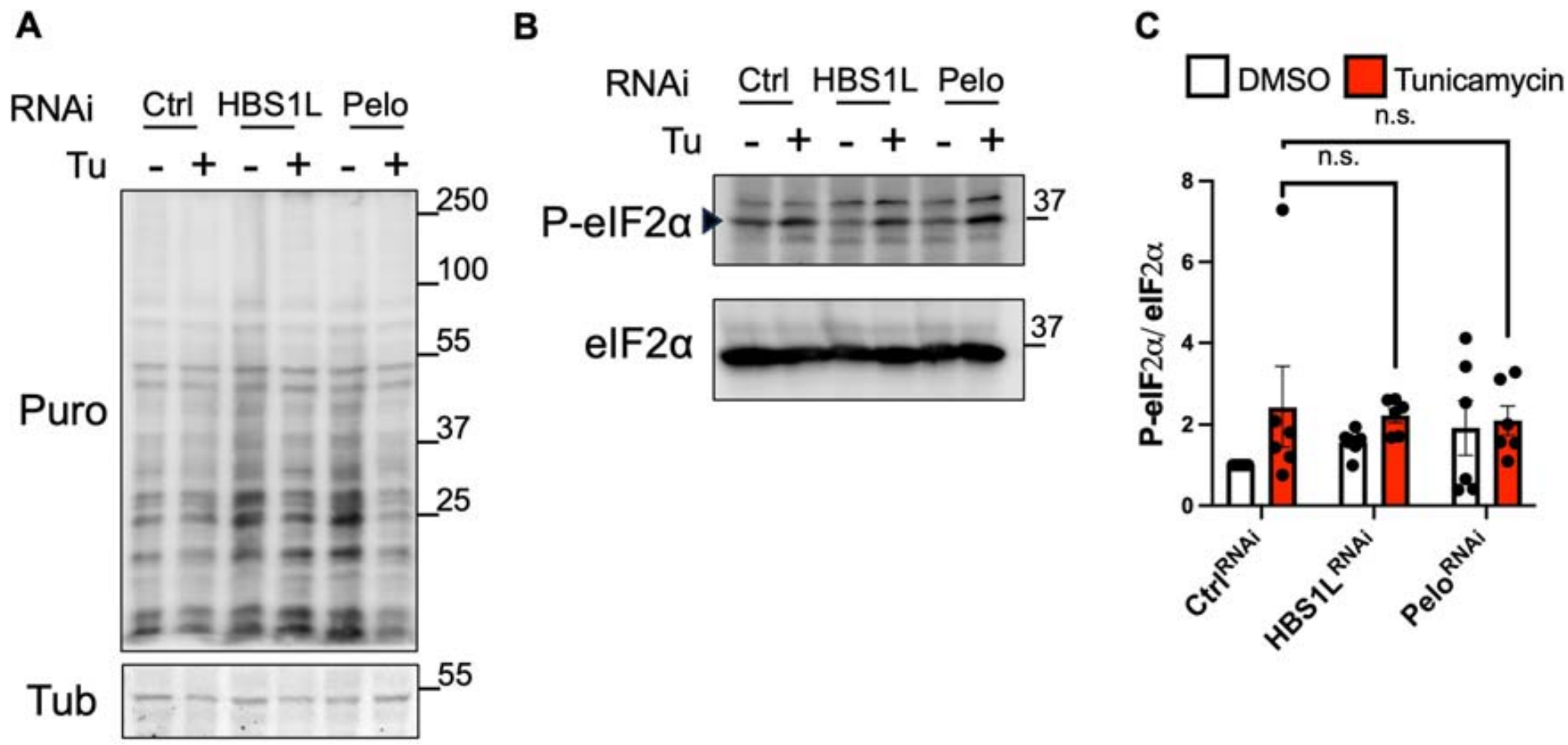

Figure EV3. Depleting HBS1L or Pelo does not have an effect on global translation or phosphorylation of eIF2α.

(**A**) Representative western blot for puromycin-incorporation in cells where HBS1L or Pelo is depleted. Quantification presented in Fig. 2F. (**B**) Western blot analysis of phospho-eIF2α (P-eIF2α) and total eIF2α in cells treated as indicated. Black arrowhead in upper panel indicates relevant P-eIF2α band. (**C**) Quantification of P-eIF2α from (**B**) normalized to total eIF2α. Data represent mean of at least five biological replicates; error bars are standard error of mean. Statistical comparisons were made using Kruskal–Wallis test with Dunn's correction. n.s. = not significant.

 © The Author(s)

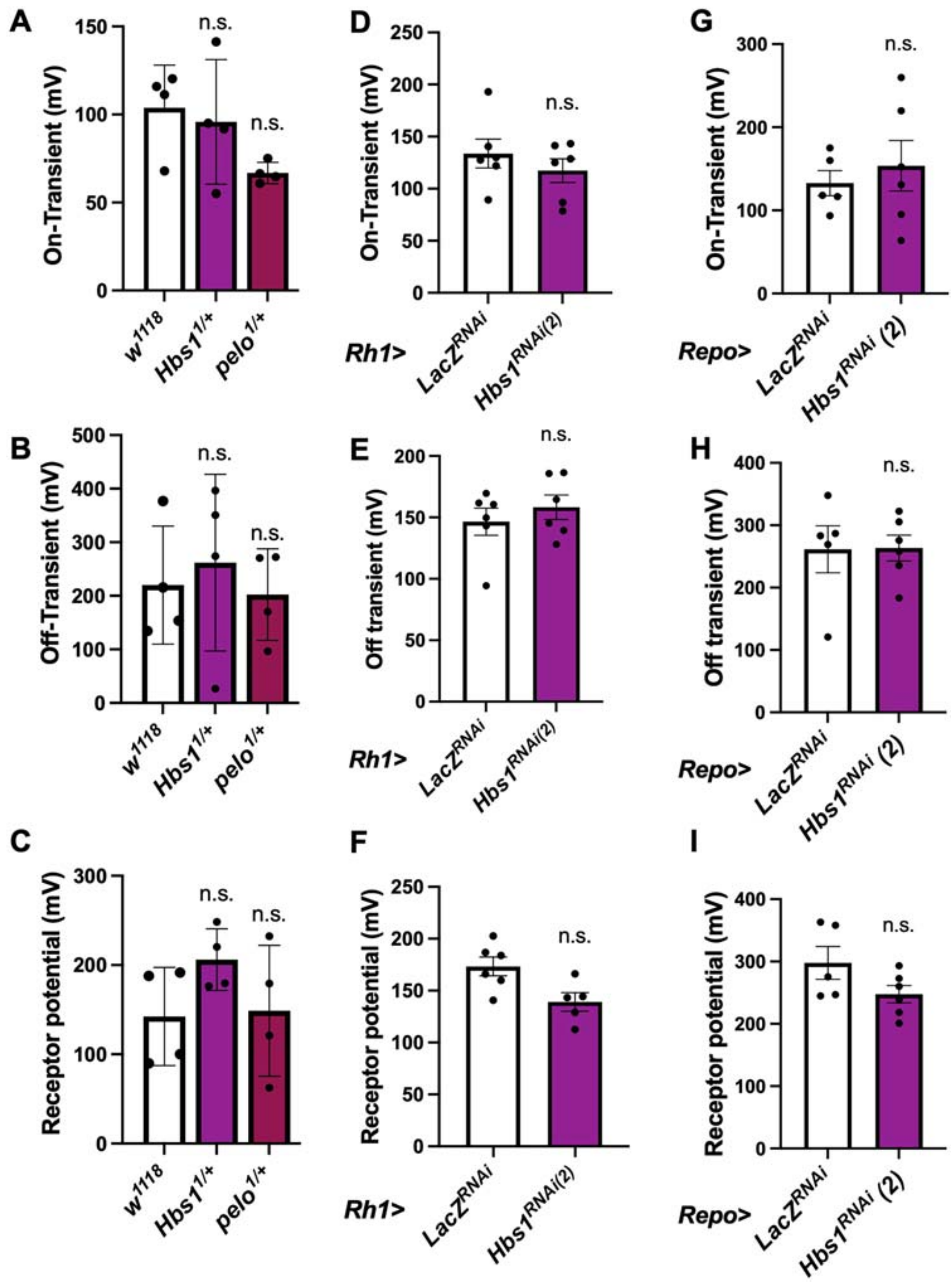

Figure EV4. Depleting *Hbs1* in photoreceptors or glia does not impact phototransduction.

(**A–C**) Quantification of on-transient (**A**), off-transient (**B**), and receptor potential (**C**) from 3–5-day-old animals from control (*w*^{1118}), *Hbs1* heterozygous (*Hbs1*^{1}/+) and *pelo* heterozygous (*pelo*^{1}/+). Each data point is the average of three technical replicates. Datasets represent the mean from at least four biological replicates; error bars are standard error of mean. Please note that the control data set here is the same as in Fig. 3 and has been replotted for convenience. Statistical comparisons were made using the Kruskal–Wallis test with Dunn's correction. (**D–I**) Quantification of on-transient (**D**, **G**), off-transient (E, H), and receptor potential (**F**, **I**) from 3–5-day-old animals where either a photoreceptor driver (*Rh1*-GAL4) or a glia driver (*Repo-GAL4*) is used to express a control *UAS-LacZ*^{RNAi} or -*Hbs1*^{RNAi}. Each data point is the average of three technical replicates. Data information: Datasets represent the mean from at least four biological replicates; error bars are standard error of mean. Statistical comparisons were made using the Mann–Whitney test. n.s. = not significant.

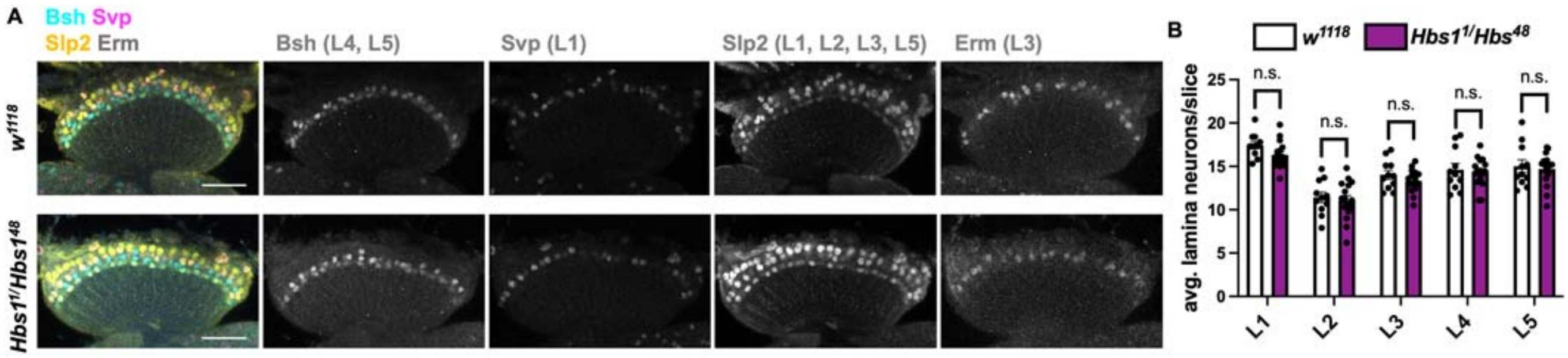

Figure EV5. Loss of *Hbs1* does not alter lamina neuron number or morphology.

(**A**) Confocal images of 3–5-day-old adult lamina from wild-type or *Hbs1* mutant animals stained markers for L1–L5 lamina neuron subtypes (Slp2 in cyan, Svp in magenta, Erm in yellow, and Bsh in gray). L1 neurons are Svp- and Slp2-positive, L2 neurons are only Slp2-positive, L3 are Erm-positive, L4 are only Bsh-positive, and L5 are Bsh- and Slp2-positive. (**B**) Counts of each lamina neuron subtype as described previously. Each data point represents one animal, data bars represent the mean of at least ten biological replicates, error bars represent standard error of mean. Statistical comparisons were made using the Mann–Whitney test. ****$p < 0.0001$, n.s. = not significant.

 © The Author(s)
